# Loss and retention of resistance genes in five species of the *Brassicaceae* family

**DOI:** 10.1186/s12870-014-0298-z

**Published:** 2014-11-01

**Authors:** Hanneke M Peele, Na Guan, Johan Fogelqvist, Christina Dixelius

**Affiliations:** Department of Plant Biology, Swedish University of Agricultural Sciences, Uppsala BioCenter, Linnean Center for Plant Biology, P.O. Box 7080, S-75007 Uppsala, Sweden

**Keywords:** *Arabidopsis thaliana*, *Brassicaceae*, CC/TIR-NB-LRR domains, Genomes, *Leptosphaeria maculans*, Resistance genes

## Abstract

**Background:**

Plants have evolved disease resistance (*R*) genes encoding for nucleotide-binding site (NB) and leucine-rich repeat (LRR) proteins with N-terminals represented by either Toll/Interleukin-1 receptor (TIR) or coiled-coil (CC) domains. Here, a genome-wide study of presence and diversification of CC-NB-LRR and TIR-NB-LRR encoding genes, and shorter domain combinations in 19 *Arabidopsis thaliana* accessions and *Arabidopsis lyrata, Capsella rubella, Brassica rapa* and *Eutrema salsugineum* are presented.

**Results:**

Out of 528 *R* genes analyzed, 12 CC-NB-LRR and 17 TIR-NB-LRR genes were conserved among the 19 *A. thaliana* genotypes, while only two CC-NB-LRRs, including *ZAR1,* and three TIR-NB-LRRs were conserved when comparing the five species. The *RESISTANCE TO LEPTOSPHAERIA MACULANS 1* (*RLM1*) locus confers resistance to the Brassica pathogen *L. maculans* the causal agent of blackleg disease and has undergone conservation and diversification events particularly in *B. rapa*. On the contrary, the *RLM3* locus important in the immune response towards *Botrytis cinerea* and *Alternaria* spp. has recently evolved in the *Arabidopsis* genus.

**Conclusion:**

Our genome-wide analysis of the *R* gene repertoire revealed a large sequence variation in the 23 cruciferous genomes. The data provides further insights into evolutionary processes impacting this important gene family.

**Electronic supplementary material:**

The online version of this article (doi:10.1186/s12870-014-0298-z) contains supplementary material, which is available to authorized users.

## Background

As sessile organisms, plants have adapted to their changing surroundings and their survival is based primarily on timely evolved immune responses. The first line of defense occurs at the plant cell surface with the recognition of conserved microbial groups such as lipopolysaccharides and peptidoglycans, commonly revered to as pathogen or microbe-associated molecular patterns (PAMPs/MAMPs). The MAMPs are recognized by cognate pattern-recognition receptors (PRRs) and trigger immediate immune responses leading to basal PAMP-triggered immunity (PTI) [1,2]. Known PRRs fall into one of two receptor classes: transmembrane receptor kinases and transmembrane receptor-like proteins, the latter of which lack any apparent internal signaling domain [3]. Notably, PRRs are components of multiprotein complexes at the plasma membrane under tight control by protein phosphatases and other regulatory proteins [4]. In a number of cases specialized pathogens are able to overcome basal PTI by either circumventing the detection of PAMPs or interfering with PTI by delaying, suppressing or reprogramming host responses via delivery of effector molecules inside host cells. As a counter mechanism, deployed intracellular resistance (R) proteins detect the presence of these effectors directly or indirectly leading to effector-triggered immunity (ETI). The RPM1-INTERACTING PROTEIN 4 (RIN4) is a well-studied key-player in the former situation [5,6], whereas direct interaction could be exemplified by the *R* genes and effectors in the rice – *Magnaporthe oryzae* pathosystem [7,8].

The plant resistance proteins are modular, that is, they consist of combinations of conserved elements some with features shared with animals reviewed by [9–11]. The majority of R proteins are typically composed of a nucleotide-binding site (NB) with a leucine-rich repeat (LRR) domain of variable length at the C-terminus. These NB-LRR proteins are divided into two classes on the basis of their N-terminal sequences consisting either of a coiled-coil (CC) sequence or of a domain that shares sequence similarity with the *Drosophila melanogaster* TOLL and human interleukin-1 receptor referred to as TIR. These blocks of conserved sequences have remained throughout evolution and can still be identified in diverse organisms of eubacteria, archaea, metazoans and bryophytes [12]. Despite this high degree of conservation, the R proteins confer resistance to a broad spectrum of plant pathogens, including viruses, bacteria, fungi, oomycetes and nematodes [13–15].

NB-encoding resistance genes have been annotated in many monocot and dicot species pioneered by *Arabidopsis thaliana* [16]. The current wealth of genomes of sequenced plant species has revealed *R* genes to be one of the largest plant gene families. In the reference genome of *A. thaliana*, 149 R-proteins harbor a LRR motif whereof 83 are composed of TIR-NB-LRR and 51 have CC-NB-LRR domains [17,18]. Several shorter proteins also are present comprising one or two domains represented by 19 TIR-NB encoding genes and 30 genes with TIR-X domains. In total, *A. thaliana* has approximately ~200 proteins with one to three *R* gene-associated protein domain combinations.

In this study we took advantage of the accelerating genome information in *A. thaliana* and performed genome-wide analyses of *R* genes in 19 *A. thaliana* genomes. We further expanded the analysis by including the genomes of the related *Arabidopsis lyrata, Capsella rubella, Brassica rapa* and *Eutrema salsugineum* species. In addition we selected two loci harboring resistance to *Brassica* fungal pathogens in order to trace down their evolutionary patterns. We found that 29 *R* genes formed a core set within *A. thaliana*, whereas as few as five *R* genes were retrieved from the genomes of the five different species. One of those five genes, the *HOPZ-ACTIVATED RESISTANCE 1* (*ZAR1*) gene known to possess novel signaling requirements is also present in other plant families within the Rosid clade. The *RESISTANCE TO LEPTOSPHAERIA MACULANS 1* (*RLM1*) locus was partly conserved in *A. lyrata* and *C. rubella* and greatly diversified in *B. rapa* and *E. salsugineum,* while the *RLM3* locus has recently evolved in the *Arabidopsis* genus. This work provides aspects on *R* gene diversity and choice of reference genotype in comparative genomic analysis.

## Results

### A core set of 29 *R* genes is present in 19 *A. thaliana* genomes

**Figure 1:**
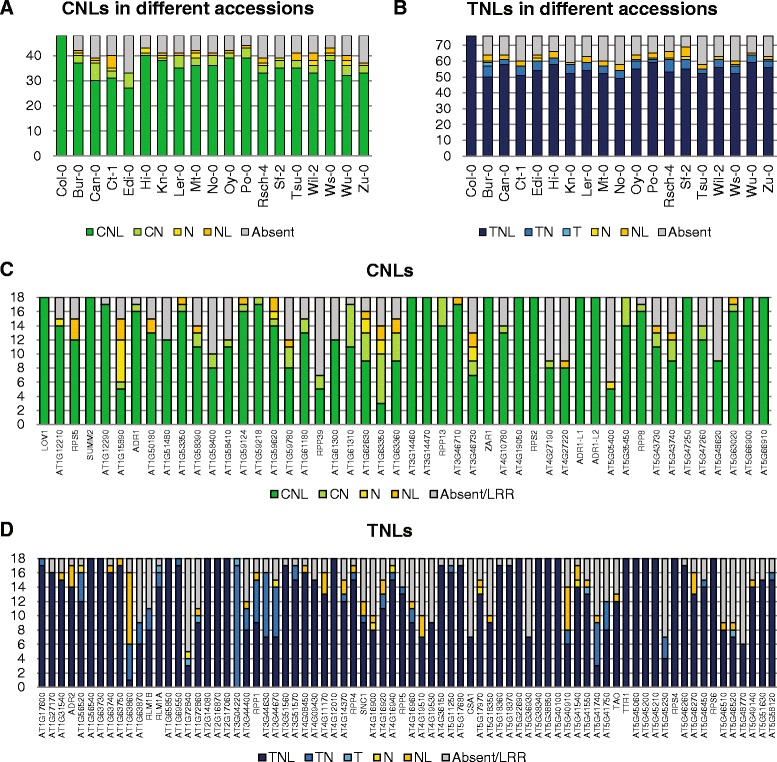
**Diversity in domain architecture of NB-LRR encoding** ***R*** **genes in 18** ***A. thaliana*** **accessions in comparison with Col-0.** In **(A)** number of genes encoding full-length or fragmented CC-NB-LRR (CNL) genes, and **(B)** number of genes encoding full-length or fragmented TIR-NB-LRR (TNL) genes. The distribution of 124 core *A. thaliana* Col-0 *R* genes in 18 *A. thaliana* accessions, with in **(C)** CNL genes and **(D)** TNL genes. For gene names, see supporting information Additional file 2: Table S2. The genes encoding only a LRR are grouped with the absent genes.

To gain insight on the level of *R* gene conservation in *A. thaliana,* we analyzed the reference genome of Col-0 and 18 additional accessions (Bur-0, Can-0, Ct-1, Edi-0, Hi-0, Kn-0, L*er*-0, Mt-0, No-0, Oy-0, Po-0, Rsch-4, Sf-2, Tsu-0, Wil-2, Ws-0, Wu-0 and Zu-0) [19]. These 18 genomes were chosen primarily for their sequence quality, high coverage, RNA sequencing data and *de novo* assembly. Pfam homology and COILS server searches on the predicted 148 NB-LRR-encoding genes [18] resulted in a reduced list of 124 *R* genes in Col-0 for further analysis, comprising 48 CC-NB-LRR (CNLs) and 76 TIR-NB-LRRs (TNLs) (Additional file 1: Table S1). Between 97 (Edi-0) to 109 (Hi-0 and Po-0) of these *R* genes were found within the genomes of the 18 newly sequenced *A. thaliana* accessions (Figure 1A, B). No additional *R* genes besides those present in Col-0 were found in the trace sequence archives of the 18 genomes.

In a comparison of the 48 CNL encoding genes in Col-0, between 27 (Edi-0) to 40 (Hi-0) were recovered in the selected accessions (Figure 1A). The protein products of the remaining genes orthologous to the CNL proteins in Col-0 were either missing one or several domains (CN, NL, N or L) or were completely absent in at least one accession (Figure 1C). Representatives of known defense-related genes that were absent included *RPS5* in Edi-0, No-0 and Sf-2, and *ADR1* in Zu-0. For gene abbreviations, see Additional file 2: Table S2. In the TNL group, the number of complete TNL genes varied between 49 (No-0) and 59 (Po-0 and Wu-0) (Figure 1B, D). Examples of missing genes were *RPP5* in Ct-1, Mt-0, Oy-0 and Wu-0, and *SNC1* in Can-0, Edi-0, No-0, Rsch-4, Tsu-0 and Wu-0.

In summary, a rather wide distribution of *R* gene repertoires was found among the 19 *A. thaliana* accessions. Out of the 124 encoding *R* genes in Col-0, 41 genes had orthologs in the other 18 accessions. However, 12 of these genes lacked one or two domains in at least one accession. For example, *RPP13* had lost its LRR domain in No-0, Rsch-4, Wil-2 and Zu-0. In the remaining core set of 12 CNL and 17 TNL encoding genes, all randomly distributed over the genome (Additional file 3: Figure S1), nine genes (*ADR1-L1*, *ADR1-L2*, *LOV1, RPS2*, *RPS4*, *RPS6*, *SUMM2*, *TTR1* and *ZAR1*), are known to be implicated in various plant defense responses.

### Five NB-LRR genes are conserved in five members of the *Brassicaceae* family

To expand the analysis on *R* genes in *A. thaliana*, we monitored possible conservation of *R* genes across lineages in *Brassicaceae* represented by *A. lyrata*, *C. rubella*, *B. rapa* and *E. salsugineum*. Pfam homology and COILS server searches identified 404 proteins with CNL or TNL architecture (Additional file 1: Table S1). The number of predicted CNL and TNL encoding genes varied greatly: *E. salsugineum* (67), *C. rubella* (75), *A. thaliana* Col-0 (124), *A. lyrata* (127), and *B. rapa* (135), numbers that do not reflect the genome sizes or number of predicted gene models in the individual species.

**Figure 2:**
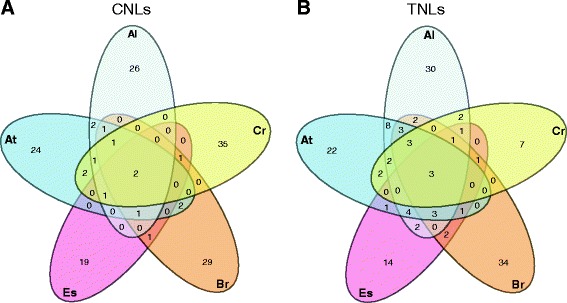
**R gene orthology between** ***A. thaliana*** **,** ***A. lyrata*** **,** ***C. rubella*** **,** ***B. rapa*** **and** ***E. salsugineum*** **.** In **(A)** the CNL orthologs and in **(B)** orthologous TNL sequences in *A. thaliana* Col-0 (*At*), *A. lyrata* (*Al*), *C. rubella* (*Cr*), *B. rapa* (*Br*) and *E. salsugineum* (*Es*). Data derived from the phylogenetic analysis (Additional file 4: Figure S2).

Orthologous sequences in the five species were identified by phylogenetic analysis of the NB domains in the CNL and TNL sequences. In the resulting phylogenetic tree, 57 clades with orthologs from at least two plant species were formed (Additional file 4: Figure S2 and Additional file 5: Table S3). Within these 57 clades, multi-copy genes from single species were also found identified as in-paralogous sequences within that specific species. The placement of the sequences outside the 57 clades was not resolved. Within the orthologous sequences a bias towards the TNL group was seen, with 52 out of 76 *A. thaliana* TNL sequences having an ortholog in one or more species, while only 17 out of 48 CNLs had an ortholog. Excluding in-paralogous genes, the highest number of orthologous sequences was identified between *A. thaliana* and *A. lyrata* (Figure 2), as concurrent with earlier findings [20,21]. From the *A. thaliana* core set of 29 genes, 7 CNL and 9 TNL genes were also found within two or more species including *ADR1-L1*, *ADR1-L2*, *RPS2*, *RPS6*, *TTR1* and *ZAR1*.

In total, two CNL clades and three TNL clades with sequences from all five species were identified. Only one of these clades (no. 5; Additional file 4: Figure S2) contained a gene implicated in defense responses, known as *ZAR1* and required for recognition of the *Pseudomonas syringae* T3SE HopZ1a effector [22]. *ZAR1* has homologs in several species within the Rosid clade as well as in *Vitis vinifera* and *Solanum* species, and in our dataset *ZAR1* was well conserved, with a *Ka*/*Ks* ratio of 0.4 supporting purifying selection. Two other genes, At5g66900 and At5g66910 were found in the same clade (no. 12; Additional file 4: Figure S2), suggesting that they were paralogous to each other and possibly have redundant functions. In this clade, *B. rapa* and *E. salsugineum* were represented with three and two genes, respectively, while there was a single gene from *A. lyrata* and *C. rubella*. Phylogenetic analysis of the CDS sequences revealed that only the At5g66900 gene was conserved among the five species (Additional file 6: Figure S3). The *RPS2* gene was earlier found in several Brassica species, including *B. montana*, *B. rapa* and *B. oleracea* [23,24]*,* and it has most likely a homolog (945467, identity of 94%) in *A. lyrata* [20]. In our dataset, the *A. thaliana RPS2* gene was also identified in *E. salsugineum* but not in *C. rubella*. However, a BLASTN homology search, revealed similarity between *RPS2* and a region annotated on the anti-sense strand as a gene without any domains in *C. rubella* (Carubv10005994m). The high similarity and identity of 88.7 suggested a possible third CNL gene being conserved among the five species.

In summary, orthology with two CNL genes (At3g50950 and At5g66900) with the possible addition of *RPS2* and three TNL genes (At4g19510, At5g45230, At5g17680) was observed in all five species. Within the 19 genomes of *A. thaliana* only the CNL genes were conserved in this particular genomic comparison. No known function has been attributed to four out of the five conserved genes, including their orthologs.

### Conservation and diversification of the *RLM1* locus

*L. maculans* is a hemitrophic fungal pathogen and the causal agent of the widespread blackleg disease of *Brassica* crops [25]. The *RLM1* locus in *A. thaliana* Col-0 was earlier identified as displaying important roles in the immune response [26] and contains seven genes with TNL architectures spanning between At1g63710 and At1g64360 (Additional file 7: Figure S4). Two genes, *RLM1A* and *RLM1B* were found to be responsible for *RLM1* activity, with *RLM1A* as the main player in the immune response [26]. No function is known for the remaining five *RLM1C*-*RLM1G* genes. Diversification in resistant loci in different accessions has been demonstrated in several cases [21,27,28] and to expand our knowledge on *RLM1*, we studied the presence and diversification of *RLM1* in our genomic data set.

**Figure 3:**
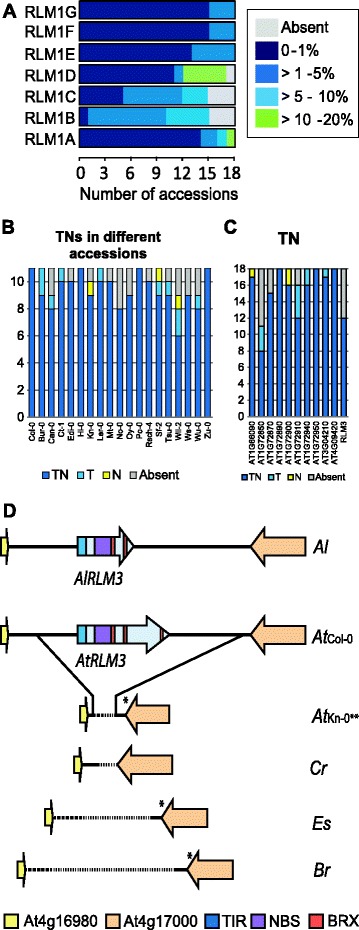
**The TNL genes within the** ***RLM1*** **locus, TN genes in 19** ***A. thaliana*** **accessions and the** ***RLM3*** **locus.** In **(A)** *p-*distance of the different *TNL* encoding proteins in the *RLM1* locus in the 19 *A. thaliana* accessions. Details on individual gene values see supporting information Additional file 9: Table S5. Domain architecture diversity of TIR-NB encoding *R* genes in 18 *A. thaliana* accessions in comparison with Col-0 with **(B)** total full-length or fragmented TIR-NB (TN) genes, and **(C)** distribution of 11 Col-0 TN proteins in 18 *A. thaliana* accessions. The genes encoding only a LRR are grouped with the absent genes. **(D)** Synteny in the *RLM3* locus between *A. thaliana* Col-0, *A. thaliana* Kn-0, *A. lyrata* (*Al*), *C. rubella* (*Cr*), *B. rapa* (*Br*) and *E. salsugineum* (*Es*). *Early stop codon; **RLM3 locus in Rsch-4, Tsu-0, Wil-2, Ws-0 and Wu-0 are identical to Kn-0.

Here, we found *RLM1A* to be present in all 18 *A. thaliana* accessions encoding all three domains in fourteen accessions (Can-0, Ct-1, Edi-0, Hi-0, L*er*-0, Mt-0, No-0, Po-0, Sf-2, Tsu-0, Wil-2, Ws-0, Wu-0 and Zu-0 (Additional file 8: Table S4). This is in agreement with their resistance phenotype [29]. In general the *RLM1A* genes in 17 accessions had very few variable sites compared to *RLM1A* in Col-0 (*p-*distance 0.2 to 0.9%). Ws-0 was atypical and diverged most with 230 variable sites in comparison to *RLM1A* in Col-0 resulting in a *p-*distance of 13.8% (Figure 3A and Additional file 9: Table S5). No *RLM1A* homologs were identified in the *A. lyrata*, *B. rapa* and *E. salsugineum* genomes. One *RLM1A* candidate was found un-annotated in the *C. rubella* genomic sequence and RNA expression data of the LRR region [30] suggests that this gene is expressed, and might have a potential role in defense responses. To support our findings, PCR amplification and sequencing of the *RLM1A* region in *A. lyrata*, *B. rapa* and *C. rubella* confirmed that only *C. rubella* has maintained *RLM1A. B. rapa* species are not known to host resistance to *L. maculans* [31] except the weedy relative *B. rapa* ssp. *sylvestris* [32,33]. In order to clarify the presence of *RLM1A* we used *RLM1A* specific primers to amplify this region in *B. napus* cv. Surpass 400 harboring resistance traits from the wild *B. rapa* relative, the gene progenitor, and for comparison, a known susceptible *B. rapa* genotype. Here, only *B. rapa* ssp. *sylvestris* contained a genomic sequence highly similar to the *RLM1A* gene of *A. thaliana* (identity 81%).

The *RLM1B* gene has a minor role in the immune response and is flanked by *RLM1C* and *RLM1D*. These three TNL genes encoded proteins lacking one or more domains in most of the 18 accessions in comparison to Col-0, especially *RLM1D* (Additional file 8: Table S4). One possible candidate orthologous to *RLM1C* was found in the genomic sequence of *C. rubella* but using the annotation of *A. thaliana* for comparison the potential gene had multiple stop codons. Similarity was found for the *RLM1B* to *RLM1C* genes in the genome of *A. lyrata*, *B. rapa* and *E. salsugineum* (Additional file 7: Figure S4). Due to the lack of orthology between species this chromosomal region seems to be under positive selection, showing a reduction of the *RLM1B* to *RLMD* genes within *A. lyrata* and *E. salsugineum*. In *B. rapa* on the contrary an expansion was observed with five TNL and one TN genes annotated to the *RLM1B-RLM1D* region, showing similarity to the *RLM1B* and *RLM1C* genes of *A. thaliana* Col-0.

The most conserved sequence within the *A. thaliana* accessions were *RLM1E, F* and *G* genes which displayed only a few modifications (*p-*distance 0.5-0.8%) (Additional file 9: Table S5). Further conservation was observed for *RLM1F* and *RLM1G* in *A. lyrata*, the latter containing two orthologs to the *RLM1F* and *RLM1G* genes with *Ka*/*Ks* ratios of 1.3 and 0.8 in comparison to *A. thaliana* Col-0. Additionally, similarity was found for *RLM1G* to the genomic region in *C. rubella* (*Ka*/*Ks* ratio of 0.7) and transcript data has previously revealed that *RLM1G* is expressed in *C. rubella* [30]. In *B. rapa,* five TNL encoding genes were found to be orthologous to *RLM1F* and *RLM1G* (clade no. 21, Additional file 4: Figure S2), but only two were found in the *RLM1* locus. The three other TNL encoding genes were located elsewhere with no synteny with the *RLM1* locus. No orthology was found for the *RLM1E* to *RLM1G* genes in *E. salsugineum*.

Overall, in the *A. thaliana* accessions the *RLM1* locus is conserved in the *RLM1E* to *RLM1G* region and appears to have experienced diversification in the *RLM1A* to *RLM1D* sequence stretch. An exception was Wu-0, in which the *RLM1* locus was highly similar to the *RLM1* locus in Col-0, with only an average *p*-distance of 0.2% (Additional file 9: Table S5). In the other four species, several of the *RLM1* genes have experienced diversification in comparison to *A. thaliana* as well as to each other. The exception is the conserved *RLM1G* in both *A. lyrata* and *C. rubella* and the *RLM1F* in *A. lyrata* while *RLM1A* was also found in *C. rubella*.

### The *RLM3* locus is unique for *A. thaliana* and *A. lyrata*

The *RLM3* gene is of importance for immune responses not only to *L. maculans* but also to *Botrytis cinerea* and *Alternaria* species [34]. The gene encodes TIR and NB domains, but lacks a LRR domain. Instead, the C-terminal end contains three copies of the DZC (disease resistance, zinc finger, chromosome condensation) or BRX domain (*brevis radix*) originally described having a role in root development [35]. In addition to *RLM3,* 18 genes in *A. thaliana* Col-0 contain TN genes without LRR domains [18]. However, *RLM3* is the only TN gene in the *A. thaliana* reference genome that contains BRX domains. To gain more insight on the TN encoding genes in *A. thaliana* Col-0, a Pfam homology and COILS server search was employed. This was designed to exclude genes with truncated TIR or NB domain, resulting in eleven TN genes (Additional file 1: Table S1). The presence of the TN encoding genes was further investigated in the 18 additional *A. thaliana* genomes.

Overall, we found between six (Wil-2) and eleven (Hi-0, Po-0 and Zu-0) genes encoding both the entire TIR and NB domain (Figure 3B). Of the eleven TN genes in Col-0, seven were present in all 18 accessions, with three encoding the complete TN. The remaining four genes encoded modifications (T or N) in at least one accession (Figure 3C). At1g72850 was absent in most accessions (Can-0, Edi-0, Mt-0, No-0, Oy-0, Wil-2 and Ws-0) and encoding only a TIR domain in Bur-0, Ct-1 and Sf-2. When we expanded the Pfam homology searches we found seven TNs in *A. lyrata*, one in *C. rubella*, sixteen in *B. rapa* and no TN encoding gene in *E. salsugineum*. Within the phylogenetic tree, five clades with orthologous proteins were identified (Additional file 4: Figure S2). None of the clades contained proteins from all four species.

A complete *RLM3* sequence was present in 13 out of 19 *A. thaliana* accessions including Col-0 and no transcripts lacking one or more domains were identified. The high *Ka*/*Ks* ratio of 2.3 suggests that *RLM3* is under positive selection in the 13 accessions. Examination of the chromosome region spanning the *RLM3* locus revealed that approximately 8,200 bp in Col-0 was completely absent in six accessions (Kn-0, Rsch-4, Tsu-0, Wil-2, Ws-0 and Wu-0), while the flanking genes; At4g16980 and At4g17000 were present (Figure 3D). The At4g17000 gene has experienced mutations and small deletions, resulting in early stop codons. The approximately 400 bp between At4g16980 and At4g17000 not found in the Col-0 genomic sequence showed minor polymorphisms between these six accessions indicating that the deletion of *RLM3* resulted from a single event.

A *RLM3-*like gene was found in *A. lyrata* (clade no. 3; Additional file 4: Figure S2) suggesting the presence of *RLM3* before the split from *A. thaliana* ~13 Mya [36]. In contrast, no *RLM3* homolog was found in the *C. rubella*, *B. rapa* and *E. salsugineum* genome sequences. To further trace a possible origin of *RLM3*, the BRX domain was used in phylogenetic analysis but no orthology could be found to sequences within the kingdom Plantae (Additional file 10: Figure S5). We conclude that *RLM3* has most likely evolved entirely within the genus of *Arabidopsis*.

## Discussion

In this report we describe a genome-wide survey of the large *R* gene family in 19 *A. thaliana* accessions and four related species in the *Brassicaceae* family. The comparisons of the *A. thaliana* accessions revealed a great variation in gene numbers and a biased loss of LRR domains. Interestingly, the Col-0 genome was the most *R* gene dense accession in the dataset. We checked for biases in the re-sequencing and gene annotation process of the additional *A. thaliana* genotypes but could not identify any obvious explanation for loss of *R* genes in these accessions. This is in line with a recent genome study comprising *de novo* assembly of 180 *A. thaliana* accessions, which revealed large variation in genome size, with 1.3-3.3 Mb of new sequences and 200–300 additional genes per genotype [37]. The differences were however found to be mainly due to 45S rDNA copies and no new *R* genes absent in Col-0 was reported.

Col-0 is a direct descendent of Col-1 and was selected from a Landsberg population based on its fertility, and vigorous plant growth [16]. The same population was used in irradiation experiments, resulting in the Landsberg *erecta* accessions (L*er*). It has now become clear that the original Landsberg population contained a mixture of slightly different genotypes, explaining the observed difference in *R* gene repertoire between Col-0 and L*er*-0. The genetic variation among *A. thaliana* accessions as observed in our dataset has a long history of being exploited for *R* gene mapping and cloning. Characterization of resistance genes to *P. syringae* (*RPM*, *RPS*) together with *RPP* genes to the oomoycete *Hyaloperonospora arabidopsidis* have been in the forefront and also advanced the understanding of interactions with pathogen effectors. The *RPP1* locus of the Ws-0 and Nd-1 accessions recognize different *H. arabidopsidis* isolates, an observation that lead to the discovery of the avirulence gene *ATR1* and six divergent alleles [38]. Sequence alignment with *ATR1* syntenic genes in *Phytophthora sojae* and *P. infestans* in turn revealed the RxLR translocation core motif, adding another dimension to the genetic makeup of host-pathogen pairs and effector biology.

Within the 18 accessions of *A. thaliana* a large number of *R* genes were missing one or more domains in comparison to Col-0, with the loss of LRR domains as the most common alteration. Modulation of the LRR sequences together with gene conversion, domain swapping and deletion events are suggested strategies for a plant to co-evolve with a pathogen. LRR domains have been identified in a diverse variety of bacterial, protist and fungal species, together representing thousands of genes [12]. Fusion of the LRR domains with the NB domain is of a more recent origin than LRR fusion with receptor-like kinases, which are seen only in the land plant lineage. The LRR domain is suggested to have evolved several times resulting in eight specific classes, which differ in sequence length and similarity within the variable segment of the LRR domain [39,40]. One of the LRR classes, referred to as Plant Specific LRRs has been shown to be under diversifying selection in several R proteins [41–44]. This type of sequence diversifications most likely reflects co-evolution with pathogen effectors, proteins known to directly or indirectly interact with the LRR motifs [7,45–47]. The importance of presence or absence of a particular LRR domain has also been demonstrated. In the absence of the *P. syringae* effector AvrPphB, the LRR domain of *RPS5* inhibits the activity of the CC and NB domains [48]. Consequently, loss of the LRR suppressor activity results in plant cell death due to constitutive *RPS5* activity. It was therefore not surprising that none of the *RPS5* homologs in our dataset lacked the LRR domain. *RPS2*, *RPS4* and *RPS6* sequences were highly conserved between accessions and the LRR domains showed low degree of polymorphisms (*Ka/Ks* ratio between 0.64 and 0.76). In case of *RPS4* the LRR domain is important for protein stability but it lacks the suppressor activity, like *RPS5* [49].

In many *A. thaliana* accessions in our dataset we found *R* genes encoding bipartite proteins, often represented by the loss of the LRR domain in comparison to Col-0. Such TN-encoding genes have been speculated to function as adapter proteins interacting with TNL proteins or with downstream signaling components [17]. For example, PBS1, an important player in the *RPS5* defense response, was found to interact with a TN protein [50]. Whether CN and TN genes in general act in protein complexes recognizing pathogen effectors remains to be demonstrated. Plant *R* genes encoding bipartite proteins also have been speculated to be part of an evolutionary reservoir in plants, allowing the formation of new genes through duplications, translocation and fusion [12,51,52]. The fusion between the TN and BRX domain in RLM3 is unique for *A. thaliana* and *A. lyrata,* possible dimerizing with other BRX domain-containing proteins, since homo- and heterodimerization capability between BRX domains of individual proteins has been shown [53]. Further, the transcription factor BRX, containing two BRX domains was shown to control the expression of a gene important in brassinolide synthesis [54] and thereby modulate both plant root and shoot growth.

In our dataset we observed a great variation in the number of unique CNL and TNL *R* genes, ranging from 33 in *E. salsugineum* to 63 in *B. rapa*. Copy number differences within different species of the *R* gene family is proposed to be driven by gene loss through pseudogenization or expansion through duplication events and subsequent divergence [12]. The five species in our dataset represent two lineages; lineage I (*Arabidopsis* and *Capsella*) and lineage II (*Brassica* and *Eutrema*), diverging at approximately 43 Mya [36,55]. Due to the close relationship between the five species, higher numbers of conserved *R* genes was expected, but no lineage-specific *R* gene repertoires were found. Comparative genomic analysis between *A. thaliana* and *B. rapa* already established orthology between several NB-LRR genes [24]. However, in our study we found eleven additional sets including orthologs to *ADR1-L1*, *ADR1-L2*, *RPP1*, *RPP13* and *ZAR1*. Out of the 528 *R* genes analyzed, only two CNLs and three TNLs were conserved in the five species. One of these, *ZAR1*, is also present in many other species within the eudicots, mainly within the Rosid clade [22]. The Rosid clade diverged from the Caryophyllales and Asterids more than 110 Mya [56] suggesting an ancient origin of the *ZAR1* gene. Recently it was shown that ZAR1 interacts with the pseudokinase ZED1 in mediating immunity to *P. syringae* [57]. This pseudokinase family is also common among flowering plants and it could be speculated that pseudokinases and ZAR1 plays a general role in basal plant defense responses not seen in the ETI response triggered by *P. syringae* in *A. thaliana.*

## Conclusions

Here, we have revealed a large variation in the *R* gene repertoire in the *A. thaliana* accessions, highlighting both the fast evolving nature of the *R* gene family but also a potential bias in the usage of a single genotype for genome comparisons. The recent advances in genome sequencing technologies enable re-sequencing of genotypes of interest for crop improvements with reasonable costs and rapid generation of molecular markers that co-segregate with traits of interest. An abundant supply of gene information from the rich genetic resources of Brassica species can therefore be foreseen along with methods for enrichment of genes of interests. Using such strategies, the number of NB-LRR genes in the potato genome was increased from 438 to 755 [58], demonstrating new avenues and breakthroughs made possible by next generation sequencing in the relatively short time that has passed since the sequencing of the first flowering plant.

## Methods

### Data sampling

The coding (CDS) and protein sequences of the *A. thaliana* Col-0 reference genome, 18 *A. thaliana* accessions, *A. lyrata, C. rubella, B. rapa* and *E. salsugineum* (previously *Thellungiella halophila*) genomes were downloaded from online databases [19,59–66]. Proteins with significant match according to the Pfam software [67] with the TIR domain (PF01582), NB-ARC (NB) domain (PF00931), and LRR domains (LRR1-5, 7–8), (PF00560, PF07723, PF07725, PF12799, PF13306, PF13504, PF13855) were selected. All proteins lacking the TIR domain were analyzed for the presence of the CC region with the COILS server using default settings and a confidence threshold >0.9 [68]. For the *A. thaliana* reference genome of Col-0 and the four species, genes encoding a TIR domain in combination of a NB and LRR (TNL) or a CC in combination with a NB and LRR (CNL) domains were selected. In the case of different isoforms, the longest transcript of each gene was included in the dataset. All protein sequences were subjected to Pfam homology and COILS server searches to identify CNL or TNL as described above for the *A. thaliana* accessions.

The *RESISTANCE TO LEPTOSPHAERIA MACULANS 1* (*RLM1*) and *RESISTANCE TO LEPTOSPHAERIA MACULANS 3* (*RLM3*) loci were selected for detailed analysis. Genomic and CDS sequences spanning two genes upstream (At1g63710) and downstream (At1g64090) of the *RLM1* locus [26] were retrieved from the TAIR10 database [16]. The CDS sequences of At1g63710 through At1g64090 in Col-0 were used to identify the corresponding chromosomal regions in *A. lyrata*, *C. rubella*, *B. rapa*, and *E. salsugineum* by BLAST search against the Phytozome database [60,69]. Similarly, the At4g16980-At4g17000 region around the *RLM3* locus (At4g16990) [34] was selected and identified in *A. lyrata*, *C. rubella*, *B. rapa*, and *E. salsugineum*. The Pfam software was used to select genes encoding a combination of TIR and NB domains (TN) in Col-0 and subsequent orthologs in the 18 *A. thaliana* accessions were identified. For the presence/absence (P/A) polymorphisms of the NB-LRR genes the definition of [70] was used. The average non-synonymous and synonymous substitutions per site ratio (*Ka*/*Ks*) for each gene were determined using the number of differences with the Nei-Gojobori distance method implemented in MEGA 5.2 [71].

### Multiple sequence alignment and phylogenetic analysis

The NB domains in the CNL and TNL proteins identified in *A. lyrata, C. rubella, B. rapa* and *E. salsugineum* genomes were aligned with ClustalW [72] using default settings and the alignment translated to nucleotides with the TranslatorX tool [73]. Poorly aligned sites were removed from the dataset using GBlocks 0.91b [74] with following settings: −b1 = 282, −b2 = 283, −b4 = 5, −b5 = h, −b6 = y. Identical proteins were reduced to one representative. A neighbor-joining tree was constructed using PAUP* 4.0β10 [75] through Geneious version 7.0.4 [76] using the GTR+G+I model with a 0.1 proportion of invariable sites and 1,000 bootstrap replicates. Proteins with a bootstrap confidence ≥70 were selected as orthologous. To further analyze parts of the resulting tree, a maximum likelihood (ML) analysis was performed using the GTR+G+I model and 1,000 bootstrap rates replicates in MEGA 5.2 [71]. Proteins with a BREVIS RADIX (BRX) domain were identified in BLASTP homology searches using a hidden Markov model (HMM) of the BRX domain sequence (PF08381). The BRX domain sequences were aligned and translated to nucleotides with translatorX and a ML tree was constructed in MEGA 5.2 using the GTR+G+I rates and 1,000 bootstrap replicates.

### Analysis of the *RLM1* and *RLM3* loci

Syntenic orthologs between *A. thaliana* Col-0, *A. lyrata*, *C. rubella*, *B. rapa*, and *E. salsugineum* were identified using the SynOrths v1.0 tool with default settings [77], by comparing all genes in the selected region between all pairs of species. Protein pairs with an *E*-value cutoff of <1e-9 were considered orthologous. All none-TNL proteins within the *RLM1* region in the different species were assigned to orthologous groups using the OrthoMCL version 2.0 server [78] followed by Pfam homology search to identify domain architecture. TNL proteins and the unannotated regions within the *RLM1* locus in the different species were aligned using ClustalW, manually inspected and classified as highly similar (≥60% aa identity) or orthologous (≥80 aa identity). The evolutionary *p*-distance (the proportion of amino acid sites at which two sequences are different divided by the total number of sites converted to percentages) between the TNL genes in the *RLM1* region of the 18 *A. thaliana* accessions [19] was calculated in comparison to Col-0 [79]. For the *RLM3* locus, the region between At4g16980-At4g17000 in *A. thaliana* Col-0, *A. lyrata, C. rubella, B. rapa* and *E. salsugineum* were aligned using ClustalW with the default settings and manually inspected.

To PCR amplify the *RLM1A* region in different species, DNA was extracted by dissolving crushed leaves of *A. lyrata,* (I2_AUT1 [80])*, C. rubella* (Cr1GR1, Samos, Greece), *B. rapa* ssp. *pekinensis* cv. ‘Granaat’, *B. napus* Surpass 400 and *B. rapa* ssp. *sylvestris* in extraction buffer (50 mM Tris, pH 7.9; 0.06 mM EDTA, pH 8; 0.62 mM Triton X-100 and 50 mM LiCl) followed by incubation at 55°C for 10 min. DNA was purified by phenol/chloroform/isoamyl alcohol (25:24:1) followed by chloroform/isoamyl alcohol (24:1), and precipitated with 3 M NaOAc (pH 5.2) and 100% ethanol. The *RLM1A* region containing part of the flanking genes (AT1G64065 and AT1G64080 in *A. thaliana*) was PCR amplified in *C. rubella* (Cr), *A. lyrata* (Al) and *B. rapa* ssp. *pekinensis* (Br) using species specific primers, Cr_Fw: GTTGTGGTTGAGATCGGTTC, Cr_Rv: TGTTGCACGAAAAGAGACAA, Al_Fw: GAACCTCCAGGGAAATGTCT, Al_Rv: CCATTGTCACTTCCGTTACC, Br_Fw: CACTTCCCCCATTAACTCCT and Br_Rv: TAAAAGCGGAGAGGGAGATT. In Surpass 400 and *B. rapa* ssp. sylvestris *RLM1A* was amplified using RLM1A_Fw3: CATCCCATTGGTCTTGATGA and RLMA_Rv3: TGGCTTTCACAAGATCACCA. The PCR products were purified using the GeneJET PCR purification kit (Thermo Scientific) followed by sequencing (Macrogen Inc. Amsterdam, the Netherlands).

## Availability of supporting data

The data supporting the results of this article are included within the article.

## Additional files

Additional file 1: Table S1. List of *R* genes in the genomes of *A. thaliana*, *A. lyrata*, *C. rubella*, *B. rapa* and *E. salsugineum*. Nomenclature is according to Phytozome or otherwise stated. Identifiers in *B. rapa* are according to [81]*. **Plant Resistance Gene Wiki [82], **Uniprot [83], §Not used in the Neighbor Joining analysis. Additional file 2: Table S2. List of *R* genes in *A. thaliana* with known function used in this study [22,26,28,34,41,84–100]. Additional file 3: Figure S1. Chromosomal distribution of conserved and selected NB-LRR genes in 19 *A. thaliana* accessions. On the right side of each chromosome the 29 conserved CNL and TNL genes are depicted together with orthologs in *A. lyrata*, *C. rubella*, *B. rapa*, and *E. salsugineum* in blue. The red genes have orthologs in the four *Brassicaceae* species but are absent in several of the *A. thaliana* accessions. Genes on the left side of the chromosomes are attributed to a defense response but were not found conserved between the 19 accessions. *R* gene information is compiled in Additional file 2: Table S2. Additional file 4: Figure S2. Phylogenetic analysis based on the NB domain in R proteins from *A. thaliana*, *A. lyrata*, *C. rubella*, *B. rapa*, and *E. salsugineum*. The neighbor joining tree was constructed using the GTR model and 1,000 bootstrap replicates. Orthologous proteins were identified in individual clades at a bootstrap value of ≥70 and are highlighted and numbered. Labeling is as follows: CNL proteins (green), TNL proteins (blue), TN proteins (light blue) and clades with bootstrap <70 (grey). The identifiers of each gene are described in Additional file 1: Table S1. Additional file 5: Table S3. Orthologous *R* genes between *A. thaliana*, *A. lyrata*, *C. rubella*, *B. rapa* and *E. salsugineum*. Additional file 6: Figure S3. Maximum likelihood analysis of ten CNL genes. The construction of the maximum likelihood tree was done using the alignment of the complete CDS sequence of the ten sequences in clade 12 (CNL) in Additional file 4: Figure S2. The GTR model was used and bootstrapping was with 1,000 replicates. The identifiers of each gene are described in Additional file 1: Table S1. Additional file 7: Figure S4. Synteny in the *RLM1* locus between five species. In (A) between *A. lyrata* (*Al*), *A. thaliana* (Col-0) (*Al*) and *C. rubella* (*Cr*) and (B) between *B. rapa* (*Cr*), *A. thaliana* (Col-0) and *E. salsugineum* (*Es*). The seven *RLM1* genes; *RLM1A* (A, At1g64070), *RLM1B*, (B, At1g63880), *RLM1C*, (C, At1g63870), *RLM1D*, (D, At1g63860), *RLM1E* (E, At1g63750), *RLM1F* (F, At1g63740) and *RLM1G* (G, At1g63730) and the other TNL encoding genes in the four species are in orange (light orange if un-annotated). The non-TNL genes are depicted in black (synteny) or white (no synteny). Synteny between genes is depicted dotted lines showing similarity between two TNL proteins with an identity of 60 or higher. Reduction in bp length is depicted by the double forward slashes. Additional file 8: Table S4. Distribution of presence and absence of gene members in the *RLM1* locus in 19 *A. thaliana* accessions. Additional file 9: Table S5.
*p-*distance of the different *TNL* encoding genes in the *RLM1* locus in the 19 *A. thaliana* accessions. Additional file 10: Figure S5. Maximum likelihood analysis of the BRX domain. The GTR model was used and bootstrapping was with 1,000 replicates. Labeling is as follows: dicots (green), monocots (dark blue), green algae (orange), moss (pink) and lycophyta (light blue). The clades consisting of BRX domains of RLM3 is highlighted.

## References

[1] Chisholm ST, Coaker G, Day B, Staskawicz BJ (2006). Host-microbe interactions: shaping the evolution of the plant immune response. Cell.

[2] Jones JDG, Dangl JL (2006). The plant immune system. Nature.

[3] Zipfel C (2008). Pattern-recognition receptors in plant innate immunity. Curr Opin Immunol.

[4] Macho AP, Zipfel C (2014). Plant PRRs and the activation of innate immune signaling. Mol Cell.

[5] Mackey D, Holt BF, Wiig A, Dangl JL (2002). RIN4 interacts with *Pseudomonas syringae* type III effector molecules and is required for RPM1-mediated resistance in *Arabidopsis*. Cell.

[6] Kim MG, da Cunha L, McFall AJ, Belkhadir Y, DebRoy S, Dangl JL, Mackey D (2005). Two *Pseudomonas syringae* type III effectors inhibit RIN4-regulated basal defense in *Arabidopsis*. Cell.

[7] Jia Y, McAdams SA, Bryan GT, Hershey HP, Valent B (2000). Direct interaction of resistance gene and avirulence gene products confers rice blast resistance. EMBO J.

[8] Kanzaki H, Yoshida K, Saitoh H, Fuijsaki K, Hirabuchi A, Alaux L, Fournier E, Tharreau D, Terauchi R (2012). Arms race co-evolution of *Magnaporthe oryzae AVR-Pik* and rice *Pik* genes driven by their physical interactions. Plant J.

[9] DeYoung BJ, Innes RW (2006). Plant NBS-LRR proteins in pathogen sensing and host defense. Nat Immunol.

[10] Dodds PN, Rathjen JP (2010). Plant immunity: towards an integrated view of plant-pathogen interactions. Nat Rev Genet.

[11] Maekawa T, Kufer TA, Schulze-Lefert P (2011). NLR functions in plant and animal immune systems: so far and yet so close. Nat Immunol.

[12] Yue JX, Meyers BC, Chen JQ, Tian D, Yang S (2012). Tracing the origin and evolutionary history of plant nucleotide-binding site-leucine-rich repeat (*NBS-LRR*) genes. New Phytol.

[13] Hammond-Kosack KE, Parker JE (2003). Deciphering plant-pathogen communication: fresh perspectives for molecular resistance breeding. Curr Opin Biotechnol.

[14] Fuller VL, Lilley CJ, Urwin PE (2008). Nematode resistance. New Phytol.

[15] Vleeshouwers VGAA, Raffaele S, Vossen JH, Champouret N, Oliva R, Segretin ME, Rietman H, Cano LM, Lokossou A, Kessel G, Pel MA, Kamoun S (2011). Understanding and exploiting late blight resistance in the age of effectors. Annu Rev Phytopathol.

[16] **TAIR10** [ftp://ftp.arabidopsis.org/home/tair/]

[17] Meyers BC, Morgante M, Michelmore RW (2002). TIR-X and TIR-NBS proteins: two new families related to disease resistance TIR-NBS-LRR proteins encoded in *Arabidopsis* and other plant genomes. Plant J.

[18] Meyers BC, Kozik A, Griego A, Kuang H, Michelmore RW (2003). Genome-wide analysis of NBS-LRR-encoding genes in Arabidopsis. Plant Cell.

[19] Gan X, Stegle O, Behr J, Steffen JG, Drewe P, Hildebrand KL, Lyngsoe R, Schultheiss SJ, Osborne EJ, Sreedharan VT, Kahles A, Bohnert R, Jean G, Derwent P, Kersey P, Belfield EJ, Harberd NP, Kemen E, Toomajian C, Kover PX, Clark RM, Rätsch G, Mott R (2011). Multiple reference genomes and transcriptomes for *Arabidopsis thaliana*. Nature.

[20] Chen Q, Han Z, Jiang H, Tian D, Yang S (2010). Strong positive selection drives rapid diversification of *R*-genes in *Arabidopsis* relatives. J Mol Evol.

[21] Guo YL, Fitz J, Schneeberger K, Ossowski S, Cao S, Weigel D (2011). Genome-wide comparison of nucleotide-binding site leucine-rich repeat-encoding genes in *Arabidopsis*. Plant Physiol.

[22] Lewis JD, Wu R, Guttman DS, Desveaux D (2010). Allele-specific virulence attenuation of the *Pseudomonas syringae* HopZ1a type III effector via the *Arabidopsis* ZAR1 resistance protein. PLoS Genet.

[23] Wroblewski T, Coulibaly S, Sadowski J, Quiros CF (2000). Variation and phylogenetic utility of the *Arabidopsis thaliana Rps2* homolog in various species of the tribe Brassiceae. Mol Phylogenet Evol.

[24] Yu J, Therim S, Zhang F, Tong C, Huang J, Cheng X, Dong C, Zhou Y, Qin R, Hua W, Liu S (2014). Genome-wide comparative analysis of NBS-encoding genes between *Brassica* species and *Arabidopsis thaliana*. BMC Genomics.

[25] Fitt BDL, Brun H, Barbetti MJ, Rimmer SR (2006). World-wide importance of phoma stem canker (*Leptosphaeria maculans* and *L. biglobosa*) on oilseed rape (*Brassica napus*). Eur J Plant Pathol.

[26] Staal J, Kaliff M, Bohman S, Dixelius C (2006). Transgressive segregation reveals two Arabidopsis TIR-NB-LRR resistance genes effective against *Leptosphaeria maculans*, causal agent of blackleg disease. Plant J.

[27] Hall SA, Allen RL, Baumber RE, Baxter LA, Fisher K, Bittner-Eddy PD, Rose LE, Holub EB, Beynon JL (2009). Maintenance of genetic variation in plants and pathogens involves complex networks of gene-for-gene interactions. Mol Plant Pathol.

[28] Goritschnig S, Krasileva KV, Dahlbeck D, Staskawicz BJ (2012). Computational prediction and molecular characterization of an oomycete effector and the cognate *Arabidopsis* resistance gene. PLoS Genet.

[29] Bohman S, Staal J, Thomma BPHJ, Wang M, Dixelius C (2004). Characterisation of an *Arabidopsis-Leptosphaeria maculans* pathosystem: resistance partially requires camalexin biosynthesis and is independent of salicylic acid, ethylene and jasmonic acid signaling. Plant J.

[30] Gos G, Slotte T, Wright SI (2012). Signatures of balancing selection are maintained at disease resistance loci following mating system evolution and a population bottleneck in the genus Capsella. BMC Evol Biol.

[31] Warwick SI, Schmidt R, Bancroft I (2011). Brassicaceae in Agriculture. Genetics and Genomics of the Brassicaceae. Volume 9.

[32] Mithen RF, Lewis BG, Heaney RK, Fenwick GR (1987). Resistance of leaves of *Brassica* species to *Leptosphaeria maculans*. Trans Brit Mycol Soc.

[33] Crouch JH, Lewis BG, Mithen RF (1994). The effect of A genome substitution on the resistance of *Brassica napus* to infection by *Leptosphaeria maculans*. Plant Breed.

[34] Staal J, Kaliff M, Dewaele E, Persson M, Dixelius C (2008). *RLM3*, a TIR domain encoding gene involved in broad-range immunity of Arabidopsis to necrotrophic fungal pathogens. Plant J.

[35] Mouchel CF, Briggs GC, Hardtke CS (2004). Natural genetic variation in *Arabidopsis* identifies *BREVIS RADIX*, a novel regulator of cell proliferation and elongation in the root. Genes Dev.

[36] Beilstein MA, Nagalingum NS, Clements MD, Manchester SR, Mathews S (2010). Dated molecular phylogenies indicate a Miocene origin for *Arabidopsis thaliana*. Proc Natl Acad Sci U S A.

[37] Long Q, Rabanal FA, Meng D, Huber CD, Farlow A, Platzer A, Zhang Q, Vilhjálmsson BJ, Korte A, Nizhynska V, Voronin V, Korte P, Sedman L, Mandáková T, Lysak MA, Seren Ü, Hellmann I, Nordborg M (2013). Massive genomic variation and strong selection in *Arabidopsis thaliana* lines from Sweden. Nat Genet.

[38] Rehmany AP, Gordon A, Rose LE, Allen RL, Armstrong MR, Whisson SC, Kamoun S, Tyler BM, Birch PRJ, Beynon JL (2005). Differential recognition of highly divergent downy mildew avirulence gene alleles by *RPP1* resistance genes from two Arabidopsis lines. Plant Cell.

[39] Kajava AV, Anisimova M, Peeters N (2008). Origin and evolution of GALA-LRR, a new member of the CC-LRR subfamily: from plants to bacteria?. PLoS ONE.

[40] Miyashita H, Kuroki Y, Kretsinger RH, Matsushima N (2013). Horizontal gene transfer of plant-specific leucine-rich repeats between plants and bacteria. Nature.

[41] McDowell JM, Dhandaydham M, Long TA, Aarts MGM, Goff S, Holub EB, Dangl JL (1998). Intragenic recombination and diversifying selection contribute to the evolution of downy mildew resistance at the *RPP8* locus of Arabidopsis. Plant Cell.

[42] Meyers BC, Shen KA, Rohani P, Gaut BS, Michelmore RW (1998). Receptor-like genes in the major resistance locus of lettuce are subject to divergent selection. Plant Cell.

[43] Ellis J, Dodds P, Pryor T (2000). Structure, function and evolution of plant disease resistance genes. Curr Opin Plant Biol.

[44] Ng A, Xavier RJ (2011). Leucine-rich repeat (LRR) proteins: integrators of pattern recognition and signaling in immunity. Autophagy.

[45] Deslandes L, Olivier J, Theulières F, Hirsch J, Feng DX, Bittner-Eddy P, Beynon J, Marco Y (2002). Resistance to *Ralstonia solanacearum* in *Arabidopsis thaliana* is conferred by the recessive *RRS1-R* gene, a member of a novel family of resistance genes. Proc Natl Acad Sci U S A.

[46] Axtell MJ, Staskawicz BJ (2003). Initiation of *RPS2*-specified disease resistance in *Arabidopsis* is coupled to the AvrRpt2-directed elimination of RIN4. Cell.

[47] Mackey D, Belkhadir Y, Alonso JM, Ecker JR, Dangl JL (2003). *Arabidopsis* RIN4 is a target of the type III virulence effector AvrRpt2 and modulates RPS2-mediated resistance. Cell.

[48] Qi D, DeYoung BJ, Innes RW (2012). Structure-function analysis of the coiled-coil and leucine-rich repeat domains of the RPS5 disease resistance protein. Plant Physiol.

[49] Zhang Y, Dorey S, Swiderski M, Jones JDG (2004). Expression of *RPS4* in tobacco induces an AvrRps4-independent HR that requires EDS1, SGT and HSP90. Plant J.

[50] Nandety RS, Caplan JL, Cavanaugh K, Perroud B, Wroblewski T, Michelmore RW, Meyers BC (2013). The role of TIR-NBS and TIR-X proteins in plant basal defense responses. Plant Physiol.

[51] Jacob F, Vernaldi S, Maekawa T (2013). Evolution and conservation of plant NLR functions. Front Immunol.

[52] Joshi RK, Nayak S (2013). Perspectives of genomic diversification and molecular recombination towards *R*-gene evolution in plants. Physiol Mol Biol Plants.

[53] Briggs GC, Mouchel CF, Hardtke CS (2006). Characterization of the plant-specific *BREVIS RADIX* gene family reveals limited genetic redundancy despite high sequence conservation. Plant Physiol.

[54] Mouchel CF, Osmont KS, Hardtke CS (2006). *BRX* mediates feedback between brassinosteroid levels and auxin signalling in root growth. Nature.

[55] Couvreur TLP, Franzke A, Al-Shehbaz IA, Bakker FT, Koch MA, Mummenhoff K (2010). Molecular phylogenetics, temporal diversification, and principles of evolution in the mustard family (*Brassicaceae*). Mol Biol Evol.

[56] Magallón S, Hilu KW, Quandt D (2013). Land plant evolutionary timeline: gene effects are secondary to fossil constraints in relaxed clock estimation of age and substitution rates. Am J Bot.

[57] Lewis JD, Lee AHY, Hassan JA, Wan J, Hurley B, Jhingree JR, Wang PW, Lo T, Youn JY, Guttman DS, Desveaux D (2013). The *Arabidopsis* ZED1 pseudokinase is required for ZAR1-mediated immunity induced by the *Pseudomonas syringae* type III effector HopZ1a. Proc Natl Acad Sci U S A.

[58] Jupe F, Witek K, Verweij W, Śliwka J, Pritchard L, Etherington GJ, Maclean D, Cock PJ, Leggett RM, Bryan GJ, Cardle L, Hein I, Jones JD (2013). Resistance gene enrichment sequencing (RenSeq) enables reannotation of the NB-LRR gene family from sequenced plant genomes and rapid mapping of resistance loci in segregating populations. Plant J.

[59] **19 genomes of*****Arabidopsis thaliana*** [http://mus.well.ox.ac.uk/19genomes/]

[60] **Phytozome** [http://www.phytozome.net/]

[61] Hu TT, Pattyn P, Bakker EG, Cao J, Cheng JF, Clark RM, Fahlgren N, Fawcett JA, Grimwood J, Gundlach H, Haberer G, Hollister JD, Ossowski S, Ottilar RP, Salamov AA, Schneeberger K, Spannagl M, Wang X, Yang L, Nasrallah ME, Bergelson J, Carrington JC, Gaut BS, Schmutz J, Mayer KFX, Van de Peer Y, Grigoriev IV, Nordborg M, Weigel D, Guo YL (2011). The *Arabidopsis lyrata* genome sequence and the basis of rapid genome size change. Nat Genet.

[62] **JGI genome portal** [http://genome.jgi-psf.org/Araly1/Araly1.download.ftp.html]

[63] Slotte T, Hazzouri KM, Ågren JA, Koenig D, Maumus F, Guo YL, Steige K, Platts AE, Escobar JS, Newman LK, Wang W, Mandáková T, Vello E, Smith LM, Henz SR, Steffen J, Takuno S, Brandvain Y, Coop G, Andolfatto P, Hu TT, Blanchette M, Clark RM, Quesneville H, Nordborg M, Gaut BS, Lysak MA, Jenkins J, Grimwood J, Chapman J (2013). The *Capsella rubella* genome and the genomic consequences of rapid mating system evolution. Nat Genet.

[64] Wang X, Wang H, Wang J, Sun R, Wu J, Liu S, Bai Y, Mun JH, Bancroft I, Cheng F, Huang S, Li X, Hua W, Wang J, Wang X, Freeling M, Pires JC, Paterson AH, Chalhoub B, Wang B, Hayward A, Sharpe AG, Park BS, Weisshaar B, Liu B, Li B, Liu B, Tong C, Song C, Duran C (2011). The genome of the mesopolyploid crop species *Brassica rapa*. Nat Genet.

[65] Yang R, Jarvis DE, Chen H, Beilstein MA, Grimwood J, Jenkins J, Shu S, Prochnik S, Xin M, Ma C, Schmutz J, Wing RA, Mitchell-Olds T, Schumaker KS, Wang X (2013). The reference genome of the halophytic plant *Eutrema salsugineum*. Front Plant Sci.

[66] Sanseverino W, Hermoso A, D’Alessandro R, Vlasova A, Andolfo G, Frusciante L, Lowy E, Roma G, Ercolano MR (2013). PRGdb 2.0: towards a community-based database model for the analysis of R-genes in plants. Nucleic Acids Res.

[67] Punta M, Coggill PC, Eberhardt RY, Mistry J, Tate J, Boursnell C, Pang N, Forslund K, Ceric G, Clements J, Heger A, Holm L, Sonnhammer ELL, Eddy SR, Bateman A, Finn RD (2012). The Pfam protein families database. Nucleic Acids Res.

[68] Lupas A, Van Dyke M, Stock J (1991). Predicting coiled coils from protein sequences. Science.

[69] Goodstein DM, Shu S, Howson R, Neupane R, Hayes RD, Fazo J, Mitros T, Dirks W, Hellsten U, Putnam N, Rokhsar DS (2011). Phytozome: a comparative platform for green plant genomics. Nucleic Acids Res.

[70] Tan S, Zhong Y, Hou H, Yang S, Tian D (2012). Variation of presence/absence genes among *Arabidopsis* populations. BMC Evol Biol.

[71] Tamura K, Peterson D, Peterson N, Stecher G, Nei M, Kumar S (2011). MEGA5: molecular evolutionary genetics analysis using maximum likelihood, evolutionary distance, and maximum parsimony methods. Mol Biol Evol.

[72] **ClustalW** [http://www.genome.jp/tools/clustalw/]

[73] Abascal F, Zardoya R, Telford MJ (2010). TranslatorX: multiple alignment of nucleotide sequences guided by amino acid translations. Nucleic Acids Res.

[74] Castresana J (2000). Selection of conserved blocks from multiple alignments for their use in phylogenetic analysis. Mol Biol Evol.

[75] Swofford DL (2002). PAUP* v4.0b8: Phylogenetic Analysis Using Parsimony * (and other Methods).

[76] **Biomatters, US** [http://www.biomatters.com/#/]

[77] Cheng F, Wu J, Fang L, Wang X (2012). Syntenic gene analysis between *Brassica rapa* and other *Brassicaceae* species. Front Plant Sci.

[78] Fischer S, Brunk BP, Chen F, Gao X, Harb OS, Iodice JB, Shanmugam D, Roos DS, Stoeckert CJ (2011). Using OrthoMCL to assign proteins to OrthoMCL-DB groups or to Cluster proteomes into new ortholog groups. Curr Protoc Bioinformatics.

[79] Nei M, Kumar S (2000). Molecular Evolution and Phylogenetics.

[80] Jørgensen MH, Ehrich D, Schmickl R, Kock MA, Brysting AK (2011). Interspecific and interploidal gene flow in Central European *Arabidopsis* (Brassicaceae). BMC Evol Biol.

[81] Mun JH, Yu HJ, Park S, Park BS (2009). Genome-wide identification of NBS-encoding resistance genes in *Brassica rapa*. Mol Genet Genomics.

[82] **Plant Resistance Gene Wiki** [http://prgdb.crg.eu/wiki/Main_Page]

[83] **Uniprot** [http://www.uniprot.org/]

[84] Grant JJ, Chini A, Basu D, Loake GJ (2003). Targeted activation tagging of the *Arabidopsis NBS-LRR* gene, *ADR1*, conveys resistance to virulent pathogens. Mol Plant Microbe Interact.

[85] Bonardi V, Tang S, Stallmann A, Roberts M, Cherkis K, Dangl JL (2011). Expanded functions for a family of plant intracellular immune receptors beyond specific recognition of pathogen effectors. Proc Natl Acad Sci U S A.

[86] Borhan MH, Gunn N, Cooper A, Gulden S, Tör M, Rimmer SR, Holub EB (2008). *WRR4* encodes a TIR-NB-LRR protein that confers broad-spectrum white rust resistance in *Arabidopsis thaliana* to four physiological races of *Albugo candida*. Mol Plant Microbe Interact.

[87] Faigón-Soverna A, Harmon FG, Storani L, Karayekov E, Staneloni RJ, Gassmann W, Más P, Casal JJ, Kay SA, Yanovsky MJ (2006). A constitutive shade-avoidance mutant implicates TIR-NBS-LRR proteins in *Arabidopsis* photomorphogenic development. Plant Cell.

[88] Lorang JM, Sweat TA, Wolpert TJ (2007). Plant disease susceptibility conferred by a “resistance” gene. Proc Natl Acad Sci U S A.

[89] Botella MA, Parker JE, Frost LN, Bittner-Eddy PD, Beynon JL, Daniels MJ, Holub EB, Jones JDG (1998). Three genes of the Arabidopsis *RPP1* complex resistance locus recognize distinct *Peronospora parasitica* avirulence determinants. Plant Cell.

[90] van der Biezen EA, Freddie CT, Kahn K, Parker JE, Jones JD (2002). Arabidopsis *RPP4* is a member of the *RPP5* multigene family of TIR-NB-LRR genes and confers downy mildew resistance through multiple signalling components. Plant J.

[91] Parker JE, Szabò V, Staskawicz BJ, Lister C, Dean C, Daniels MJ, Jones JDG (1993). Phenotypic characterization and molecular mapping of the *Arabidopsis thaliana* locus *RPP5*, determining disease resistance to *Peronospora parasitica*. Plant J.

[92] Bittner-Eddy P, Can C, Gunn N, Pinel M, Tör M, Crute I, Holub EB, Beynon J (1999). Genetic and physical mapping of the *RPP13* locus, in Arabidopsis, responsible for specific recognition of several *Peronospora parasitica* (downy mildew) isolates. Mol Plant Microbe Interact.

[93] Mindrinos M, Katagiri F, Yu GL, Ausubel FM (1994). The A. thaliana disease resistance gene RPS2 encodes a protein containing a nucleotide-binding site and leucine-rich repeats. Cell.

[94] Gassmann W, Hinsch ME, Staskawicz BJ (1999). The Arabidopsis *RPS4* bacterial-resistance gene is a member of the TIR-NBS-LRR family of disease-resistance genes. Plant J.

[95] Warren RF, Henk A, Mowery P, Holub E, Innes RW (1998). A mutation within the leucine-rich repeat domain of the Arabidopsis disease resistance gene *RPS5* partially suppresses multiple bacterial and downy mildew resistance genes. Plant Cell.

[96] Kim SH, Kwon SI, Saha D, Anyanwu NC, Gassmann W (2009). Resistance to the *Pseudomonas syringae* effector HopA1 is governed by the TIR-NBS-LRR protein RPS6 and is enhanced by mutations in *SRFR1*. Plant Physiol.

[97] Stokes TL, Kunkel BN, Richards EJ (2002). Epigenetic variation in *Arabidopsis* disease resistance. Genes Dev.

[98] Zhang Z, Wu Y, Gao M, Zhang J, Kong Q, Liu Y, Ba H, Zhou J, Zhang Y (2012). Disruption of PAMP-induced MAP kinase cascade by a *Pseudomonas syringae* effector activates plant immunity mediated by the NB-LRR protein SUMM2. Cell Host Microbe.

[99] Eitas TK, Nimchuk ZL, Dangl JL (2008). *Arabidopsis* TAO1 is a TIR-NB-LRR protein that contributes to disease resistance induced by the *Pseudomonas syringae* effector AvrB. Proc Natl Acad Sci U S A.

[100] Nam M, Koh S, Kim SU, Domier LL, Jeon JH, Kim HG, Lee SH, Bent AF, Moon JS (2011). *Arabidopsis TTR1* causes LRR-dependent lethal systemic necrosis, rather than systemic acquired resistance, to tobacco ringspot virus. Mol Cells.

