# Supplementary material for: Loss and retention of resistance genes in five species of the Brassicaceae family
Source: BMC Plant Biol. 2014 Nov 1;14:298. doi: 10.1186/s12870-014-0298-z (PMC4232680; doi:10.1186/s12870-014-0298-z)
Supplement: Additional file 1: Table S1. — List of R genes in the genomes of A. thaliana, A. lyrata, C. rubella, B. rapa and E. salsugineum. Nomenclature is according to Phytozome or otherwise stated. Identifiers in B. rapa are according to [81]. *Plant Resistance Gene Wiki [82], **Uniprot [83], §Not used in the Neighbor Joining analysis. [file 12870_2014_298_MOESM1_ESM.docx]

**Table S1** List of *R* genes in the genomes of *A. thaliana*, *A. lyrata*, *C. rubella*, *B. rapa* and Eutrema salsugineum. Nomenclature is according to Phytozome or otherwise stated. Identifiers in B. rapa are according to [81]. * Plant Resistance Gene Wiki [82], ** Uniprot [83], § Not used in the Neighbor Joining analysis.

| **CNL** | ***A. thaliana*** | ***A.lyrata*** | ***C. rubella*** | ***B. rapa*** | ***E. salsugineum*** |
| --- | --- | --- | --- | --- | --- |
| 1 | AT1G10920 | 330052 | Carubv10012293m | - | Thhalv10002924m |
| 2 | AT1G12210 | 338119 | Carubv10019764m | D1GEF5** | Thhalv10023256m |
| 3 | AT1G12220 | 907240 | - | Bra036995 | Thhalv10006759m |
| 4 | AT1G12280 | 338250 | Carubv10003836m | D1GEH8** | Thhalv10023263m |
| 5 | AT1G12290 | 920497 | Carubv10022460m | D1GEH9** | Thhalv10023246m |
| 6 | AT1G15890 | 891371 | Carubv10019789m | Bra036845 | Thhalv10000112m |
| 7 | AT1G33560§ | 493640 | Carubv10019775m | D1GEI8** | Thhalv10023255m |
| 8 | AT1G50180 | 497178 | Carubv10019773m | Bra020568 | - |
| 9 | AT1G51480 | 915860 | Carubv10019813m | D1GEJ5** | Thhalv10023286m |
| 10 | AT1G53350§ | 351902 | Carubv10012171m | D1GEA8** | Thhalv10023301m |
| 11 | AT1G58390 | 945467 | Carubv10027797m | D1GEB6** | Thhalv10023248m |
| 12 | AT1G58400 | 492076 | Carubv10025875m | Bra019063 | Thhalv10023266m |
| 13 | AT1G58410 | PRGDB00150455* | Carubv10008267m | - | Thhalv10023258m |
| 14 | AT1G59124 | 923884 | Carubv10025854m | Bra013213 | Thhalv10028114m |
| 15 | AT1G59218 | 910841 | Carubv10027796m | D1GED1** | Thhalv10028060m |
| 16 | AT1G59620 | 494924 | Carubv10004109m | Bra027866 | Thhalv10017979m |
| 17 | AT1G59780 | 887877 | Carubv10028494m | Bra018863 | Thhalv10000769m |
| 18 | AT1G61180 | 894148 | Carubv10019753m | Bra018245 | Thhalv10000773m |
| 19 | AT1G61190 | 907577 | Carubv10000162m | Bra026977 | Thhalv10024353m |
| 20 | AT1G61300 | 914885 | Carubv10021618m | D1GEB5** | Thhalv10024315m |
| 21 | AT1G61310 | 907505 | Carubv10019741m | M4CE64** | Thhalv10012605m |
| 22 | AT1G62630 | 474150 | Carubv10022028m | Bra013947 | Thhalv10023237m |
| 23 | AT1G63350 | 485448 | Carubv10019818m | Bra026978 | Thhalv10023289m |
| 24 | AT1G63360 | PRGDB00150586* | Carubv10019767m | Bra026094 | Thhalv10010117m |
| 25 | AT3G14460 | PRGDB00149402* | Carubv10019761m | Bra016785 | Thhalv10020099m |
| 26 | AT3G14470 | PRGDB00150179* | Carubv10019807m | Bra019754 | Thhalv10003662m |
| 27 | AT3G46530 | PRGDB00150679* | Carubv10022288m | Bra016782 | Thhalv10003136m |
| 28 | AT3G46710 | 473526 | Carubv10019750m | Bra026368 | Thhalv10024259m |
| 29 | AT3G46730 | 944602 | Carubv10019766m | Bra016781 | - |
| 30 | AT3G50950 | - | Carubv10019824m | Bra034631 | - |
| 31 | AT4G10780 | 334514 | Carubv10019740m | Bra018448 | - |
| 32 | AT4G19050 | - | Carubv10021922m | Bra037451 | - |
| 33 | AT4G26090 | 312255_Nterminus | Carubv10008237m | Bra037448 | - |
| 34 | AT4G27190 | 312255_Cterminus | Carubv10016680m | Bra005273 | - |
| 35 | AT4G27220 | 915866 | Carubv10015458m | Bra005271 | - |
| 36 | AT4G33300 | 496910 | Carubv10012877m | Bra035424 | - |
| 37 | AT5G04720 | 915590 | Carubv10000246m | Bra029405 | - |
|  | ***A. thaliana*** | ***A.lyrata*** | ***C. rubella*** | ***B. rapa*** | ***E. salsugineum*** |
| 38 | AT5G05400 | 492950 | Carubv10027797m | Bra036206 | - |
| 39 | AT5G35450 | 890263 | Carubv10004147m | D1GEE0** | - |
| 40 | AT5G43470 | - | Carubv10006587m | Bra027332 | - |
| 41 | AT5G43730 | - | Carubv10004009m | D1GEG4** | - |
| 42 | AT5G43740 | - | Carubv10008215m | Bra037139 | - |
| 43 | AT5G47250 | - | Carubv10027991m | Bra009882 | - |
| 44 | AT5G47260 | - | Carubv10020008m | Bra026924 | - |
| 45 | AT5G48620 | - | Carubv10022134m | D1GEB9** | - |
| 46 | AT5G63020 | - | - | Bra027598 | - |
| 47 | AT5G66900 | - | Carubv10019869m | Bra026682 | - |
| 48 | AT5G66910 | - | - | Bra034556 | - |
| **TNL** |  |  |  |  |  |
| 1 | AT1G17600 | 893040 | Carubv10021911m | Bra035103 | Thhalv10023235m |
| 2 | AT1G27170 | 887645 | Carubv10019662m | Bra022850 | Thhalv10023861m |
| 3 | AT1G31540 | 916001 | Carubv10022170m | D1GEF9** | Thhalv10011222m |
| 4 | AT1G56510 | 887642 | Carubv10019665m | - | Thhalv10012489m |
| 5 | AT1G56520 | 887641 | Carubv10027634m | - | Thhalv10024211m |
| 6 | AT1G56540 | 908051 | - | D1GEG2** | Thhalv10024295m |
| 7 | AT1G63730 | 891930 | Carubv10004008m | - | Thhalv10024234m |
| 8 | AT1G63740 | 891933 | Carubv10025753m | D1GEG5** | Thhalv10019540m |
| 9 | AT1G63750 | 891936 | Carubv10025432m | D1GEG6** | Thhalv10000759m |
| 10 | AT1G63860 | 896915 | Carubv10004054m | - | - |
| 11 | AT1G63870 | 915422_Nterminus | Carubv10004053m | Bra009141 | Thhalv10012477m |
| 12 | AT1G63880 | 897229 | Carubv10020469m | Bra002117 | Thhalv10022404m |
| 13 | AT1G64070 | 897230 | Carubv10003908m | Bra024651 | Thhalv10001229m |
| 14 | AT1G65850 | 891298 | Carubv10019790m | D1GEH4** | Thhalv10000753m |
| 15 | AT1G69550 | 915002 | Carubv10019730m | D1GEH5** | Thhalv10019465m |
| 16 | AT1G72840 | 915006 | Carubv10008094m | D1GEH6** | Thhalv10000023m |
| 17 | AT1G72860 | 948663 | Carubv10007584m | D1GEI0** | Thhalv10027634m |
| 18 | AT2G14080 | 915423 | Carubv10025757m | Bra023670 | Thhalv10000695m |
| 19 | AT2G16870 | 915425 | Carubv10025767m | Bra012540 | - |
| 20 | AT2G17060 | 494325 | Carubv10022536m | Bra012541 | Thhalv10018052m |
| 21 | AT3G04220 | 896835 | Carubv10004072m | D1GEI9** | Thhalv10023230m |
| 22 | AT3G44400 | 916055 | - | Bra010551 | Thhalv10023951m |
| 23 | AT3G44480 | 903839 | Carubv10025792m | Bra000759 | Thhalv10023884m |
| 24 | AT3G44630 | 876227 | - | - | Thhalv10019585m |
| 25 | AT3G44670 | 489751 | Carubv10022539m | - | Thhalv10027631m |
| 26 | AT3G51560 | 494346 | Carubv10007461m | D1GEJ4** | Thhalv10028092m |
| 27 | AT3G51570 | 915424 | Carubv10002826m | Bra023647 | Thhalv10023232m |
| 28 | AT4G08450 | 915416 | Carubv10006430m | - | Thhalv10012455m |
| 29 | AT4G09430 | 890529 | Carubv10002693m | - | Thhalv10024348m |
| 30 | AT4G11170 | 914947 | Carubv10004129m | - | Thhalv10023218m |
| 31 | AT4G12010 | 914883 | Carubv10006829m | - | Thhalv10006573m |
| 32 | AT4G14370 | 915422_Cterminus | Carubv10007493m | D1GEB1** | Thhalv10010076m |
|  | ***A. thaliana*** | ***A.lyrata*** | ***C. rubella*** | ***B. rapa*** | ***E. salsugineum*** |
| 33 | AT4G16860 | 907036 | Carubv10027816m | D1GEB2** | Thhalv10000750m |
| 34 | AT4G16890 | 890967 | - | Bra030994 | Thhalv10012555m |
| 35 | AT4G16900 | 474976 | - | Bra019273 | - |
| 36 | AT4G16920 | 907024 | - | - | Thhalv10000021m |
| 37 | AT4G16940 | 480565 | - | - | Thhalv10001215m |
| 38 | AT4G16950 | 907275 | - | - | Thhalv10000747m |
| 39 | AT4G16960§ | 907021 | - | - | Thhalv10000751m |
| 40 | AT4G19510 | 907475 | - | - | Thhalv10024441m |
| 41 | AT4G19530 | 891510 | - | - | Thhalv10010075m |
| 42 | AT4G36150 | 891488 | - | D1GEC9** | Thhalv10001238m |
| 43 | AT5G11250 | 900537 | - | - | Thhalv10027276m |
| 44 | AT5G17680 | 891508 | - | D1GED3** | - |
| 45 | AT5G17880 | 891573 | - | Bra006556 | - |
| 46 | AT5G17970 | 891572 | - | Bra036790 | - |
| 47 | AT5G18350 | 891511 | - | D1GED9** | - |
| 48 | AT5G18360 | 893714 | - | D1GEE3** | - |
| 49 | AT5G18370 | 893757 | - | Bra017542 | - |
| 50 | AT5G22690 | 947767 | - | D1GEE6** | - |
| 51 | AT5G36930 | 471969 | - | - | - |
| 52 | AT5G38340 | 908896 | - | D1GEE9** | - |
| 53 | AT5G38850 | 908894 | - | Bra010663 | - |
| 54 | AT5G40100 | 488633 | - | Bra010589 | - |
| 55 | AT5G40910 | 912308 | - | Bra010496 | - |
| 56 | AT5G41540 | 912305 | - | Bra010590 | - |
| 57 | AT5G41550 | 921508 | - | Bra017807 | - |
| 58 | AT5G41740 | 333042 | - | Bra006146 | - |
| 59 | AT5G41750 | PRGDB00150004* | - | Bra027510 | - |
| 60 | AT5G44510 | PRGDB00150273* | - | Bra036799 | - |
| 61 | AT5G44870 | PRGDB00149758* | - | Bra038872 | - |
| 62 | AT5G45060 | PRGDB00149759* | - | Bra036417 | - |
| 63 | AT5G45200 | PRGDB00149757* | - | Bra019409 | - |
| 64 | AT5G45210 | PRGDB00149853* | - | Bra019410 | - |
| 65 | AT5G45230 | 485509 | - | Bra020861 | - |
| 66 | AT5G45250 | 948472 | - | Bra001162 | - |
| 67 | AT5G46260 | 915598 | - | Bra001161 | - |
| 68 | AT5G46270 | 494475 | - | Bra001160 | - |
| 69 | AT5G46450 | 948566 | - | Bra006452 | - |
| 70 | AT5G46470 | 948495 | - | Bra002153 | - |
| 71 | AT5G46510 | 915593 | - | Bra040210 | - |
| 72 | AT5G46520 | 915659 | - | Bra003997 | - |
| 73 | AT5G48770 | 893705 | - | Bra034079 | - |
| 74 | AT5G49140 | 320248 | - | Bra022071 | - |
| 75 | AT5G51630 | 915612 | - | Bra022037 | - |
| 76 | AT5G58120 | 888440 | - | Bra022039 | - |
| 77 | - | 932480 | - | Bra025639 | - |
|  | ***A. thaliana*** | ***A.lyrata*** | ***C. rubella*** | ***B. rapa*** | ***E. salsugineum*** |
| 78 | - | 915620 | - | Bra027598 | - |
| 79 | - | 330904 | - | Bra012688 | - |
| 80 | - | 494460 | - | Bra027790 | - |
| 81 | - | 915600 | - | Bra027791 | - |
| 82 | - | 333306 | - | Bra021754 | - |
| 83 | - | 890225 | - | Bra032045 | - |
| 84 | - | 327329 | - | Bra027778 | - |
| 85 | - | 323710 | - | Bra027779 | - |
| 86 | - | 915658 | - | Bra027772 | - |
| 87 | - | 941306 | - | Bra027775 | - |
| 88 | - | 915611 | - | Bra027780 | - |
| 89 | - | 925117 | - | Bra025493 | - |
| 90 | - | 890226 | - | Bra025467 | - |
| 91 | - | - | - | Bra028500 | - |
| 92 | - | - | - | Bra028499 | - |
| 93 | - | - | - | Bra003867 | - |
| 94 | - | - | - | Bra008053 | - |
| 95 | - | - | - | Bra016594 | - |
| 96 | - | - | - | Bra023631 | - |
| 97 | - | - | - | Bra013959 | - |
| 98 | - | - | - | Bra018037 | - |
| 99 | - | - | - | Bra035126 | - |
| 100 | - | - | - | Bra016314 | - |
| 101 | - | - | - | Bra024652 | - |
| 102 | - | - | - | Bra027599 | - |
| 103 | - | - | - | Bra021953 | - |
| 104 | - | - | - | Bra021956 | - |
| 105 | - | - | - | D6RS99 | - |
| 106 | - | - | - | Bra029431 | - |
| 107 | - | - | - | Bra021952 | - |
| 108 | - | - | - | Bra013400 | - |
| **TN** |  |  |  |  |  |
| 1 | AT1G66090 | 889979 | Carubv10019685m | Bra006487 | - |
| 2 | AT1G72850 | 916056 | - | D1GEI3** | - |
| 3 | AT1G72870 | 915427 | - | D1GEI6** | - |
| 4 | AT1G72890 | 493178 | - | D1GEA5** | - |
| 5 | AT1G72900 | 893712 | - | D1GEA6** | - |
| 6 | AT1G72910 | 948501 | - | Bra030995 | - |
| 7 | AT1G72940 | 896621 | - | D1GEC0** | - |
| 8 | AT1G72950 | - | - | Bra016029 | - |
| 9 | AT3G04210 | - | - | D1GEE8** | - |
| 10 | AT4G09420 | - | - | D1GEF3** | - |
| 11 | AT4G16990 | - | - | PRGDB00200894* | - |
| 12 | - | - | - | Bra033939 | - |
| 13 | - | - | - | Bra036432 | - |
|  | ***A. thaliana*** | ***A.lyrata*** | ***C. rubella*** | ***B. rapa*** | ***E. salsugineum*** |
| 14 | - | - | - | M4CV15** | - |
| 15 | - | - | - | M4CI33** | - |
| 16 | - | - | - | M4CV11** | - |
| 17 | - | - | - | D1GEE8** | - |
