# Supplementary material for: Loss and retention of resistance genes in five species of the Brassicaceae family
Source: BMC Plant Biol. 2014 Nov 1;14:298. doi: 10.1186/s12870-014-0298-z (PMC4232680; doi:10.1186/s12870-014-0298-z)
Supplement: Additional file 2: Table S2. — List of R genes in A. thaliana with known function used in this study [22,26,28,34,41,84–100]. [file 12870_2014_298_MOESM2_ESM.docx]

**Table S2** List of *R* genes in *A. thaliana* with known function used in this study.

| **Name** | **Abbreviation** | **Reference** |
| --- | --- | --- |
| *ACTIVATED DISEASE RESISTANCE 1* | *ADR1* | [84] |
| *ACTIVATED DISEASE RESISTANCE 1-LIKE 1* | *ADR1-L1* | [85] |
| *ACTIVATED DISEASE RESISTANCE 1-LIKE 2* | *ADR1-L2* | [85] |
| *ACTIVATED DISEASE RESISTANCE 2* | *ADR2* | [86] |
| *CONSTITUTIVE SHADE-AVOIDANCE 1* | *CSA1* | [87] |
| *LOCUS ORCHESTRATING VICTORIN EFFECTS 1* | *LOV1* | [88] |
| *RESISTANCE AGAINST L. MACULANS 1A* | *RLM1A* | [26] |
| *RESISTANCE AGAINST L. MACULANS 1B* | *RLM1B* | [26] |
| *RESISTANCE AGAINST L. MACULANS 3* | *RLM3* | [34] |
| *RECOGNITION OF P. PARASITICA 1* | *RPP1* | [89] |
| *RECOGNITION OF P. PARASITICA 4* | *RPP4* | [90] |
| *RECOGNITION OF P. PARASITICA 5* | *RPP5* | [91] |
| *RECOGNITION OF P. PARASITICA 8* | *RPP8* | [41] |
| *RECOGNITION OF P. PARASITICA 13* | *RPP13* | [92] |
| *RECOGNITION OF P. PARASITICA 39* | *RPP39* | [28] |
| *RESISTANT TO P. SYRINGAE 2* | *RPS2* | [93] |
| *RESISTANT TO P. SYRINGAE 4* | *RPS4* | [94] |
| *RESISTANT TO P. SYRINGAE 5* | *RPS5* | [95] |
| *RESISTANT TO P. SYRINGAE 6* | *RPS6* | [96] |
| *SUPPRESSOR OF NPR1-1, CONSTITUTIVE 1* | *SNC1* | [97] |
| *SUPPRESSOR OF MKK1 MKK2 2* | *SUMM2* | [98] |
| *TARGET OF AVRB OPERATION1* | *TAO* | [99] |
| *TOLERANCE TO TOBACCO RINGSPOT VIRUS* *1* | *TTR1* | [100] |
| *HOPZ ACTIVATED RESISTANCE 1* | *ZAR1* | [22] |
