# Supplementary figures and images for: Loss and retention of resistance genes in five species of the Brassicaceae family
Source: BMC Plant Biol. 2014 Nov 1;14:298. doi: 10.1186/s12870-014-0298-z (PMC4232680; doi:10.1186/s12870-014-0298-z)

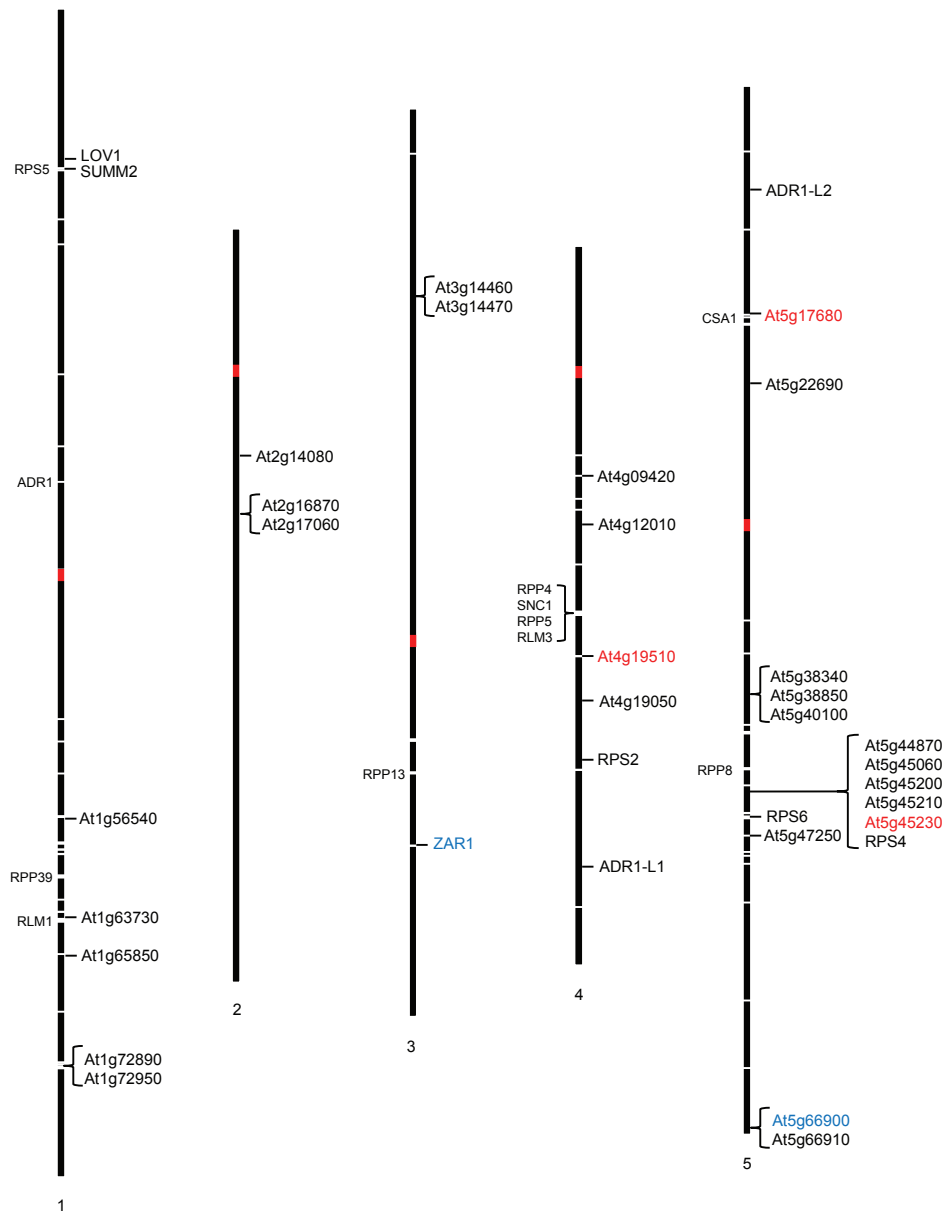

Supplement: Additional file 3: Figure S1. — Chromosomal distribution of conserved and selected NB-LRR genes in 19 A. thaliana accessions. On the right side of each chromosome the 29 conserved CNL and TNL genes are depicted together with orthologs in A. lyrata, C. rubella, B. rapa, and E. salsugineum in blue. The red genes have orthologs in the four Brassicaceae species but are absent in several of the A. thaliana accessions. Genes on the left side of the chromosomes are attributed to a defense response but were not found conserved between the 19 accessions. R gene information is compiled in Additional file 2: Table S2. [file 12870_2014_298_MOESM3_ESM.pdf]

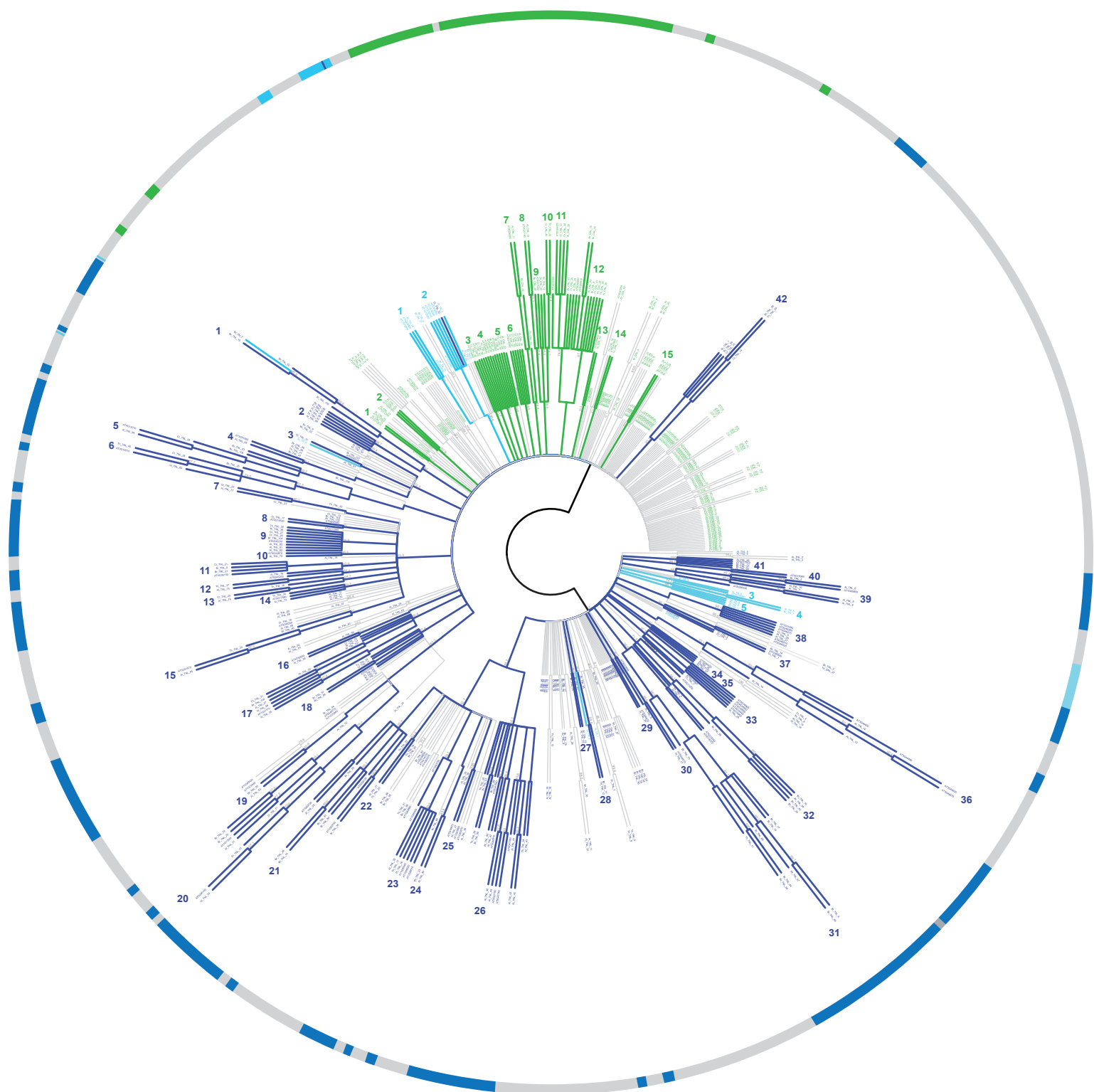

Supplement: Additional file 4: Figure S2. — Phylogenetic analysis based on the NB domain in R proteins from A. thaliana, A. lyrata, C. rubella, B. rapa, and E. salsugineum. The neighbor joining tree was constructed using the GTR model and 1,000 bootstrap replicates. Orthologous proteins were identified in individual clades at a bootstrap value of ≥70 and are highlighted and numbered. Labeling is as follows: CNL proteins (green), TNL proteins (blue), TN proteins (light blue) and clades with bootstrap <70 (grey). The identifiers of each gene are described in Additional file 1: Table S1. [file 12870_2014_298_MOESM4_ESM.pdf]

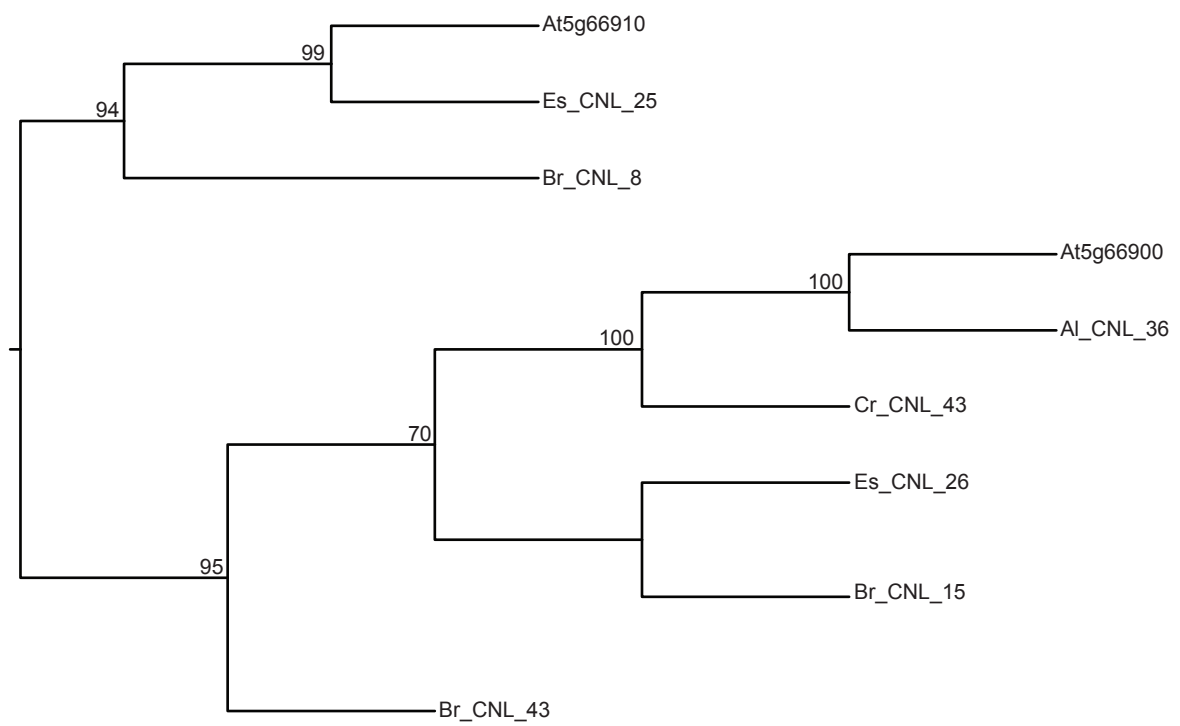

Supplement: Additional file 6: Figure S3. — Maximum likelihood analysis of ten CNL genes. The construction of the maximum likelihood tree was done using the alignment of the complete CDS sequence of the ten sequences in clade 12 (CNL) in Additional file 4: Figure S2. The GTR model was used and bootstrapping was with 1,000 replicates. The identifiers of each gene are described in Additional file 1: Table S1. [file 12870_2014_298_MOESM6_ESM.pdf]

(a)

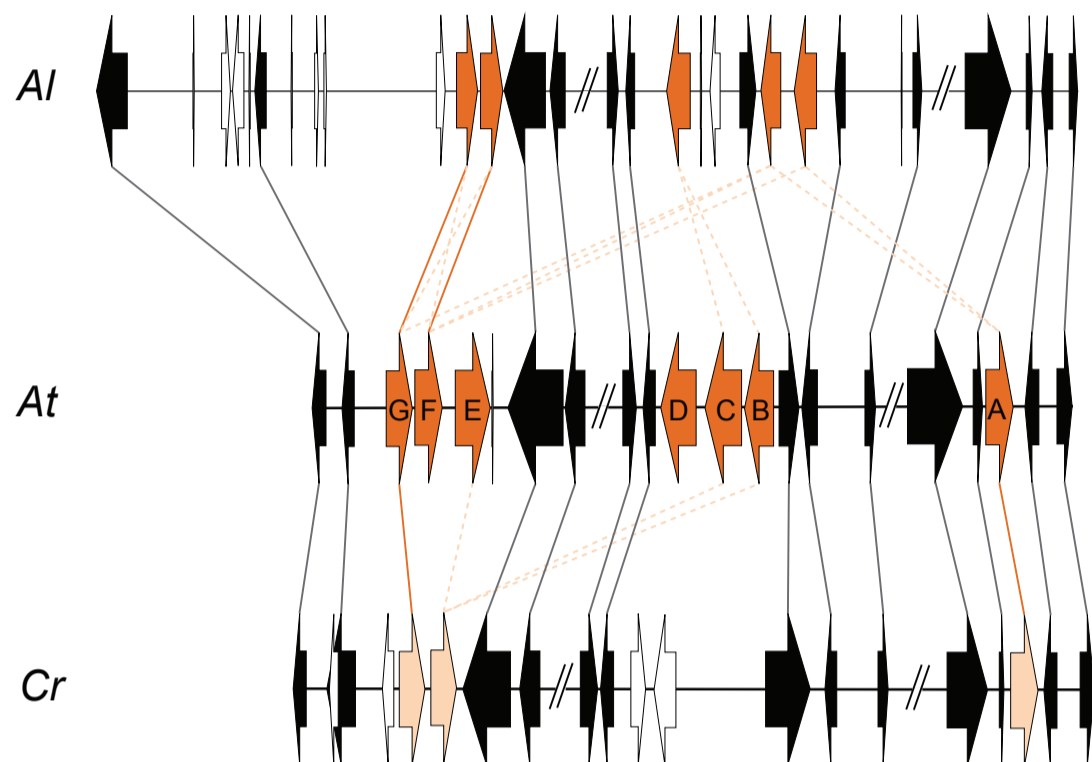

(b)

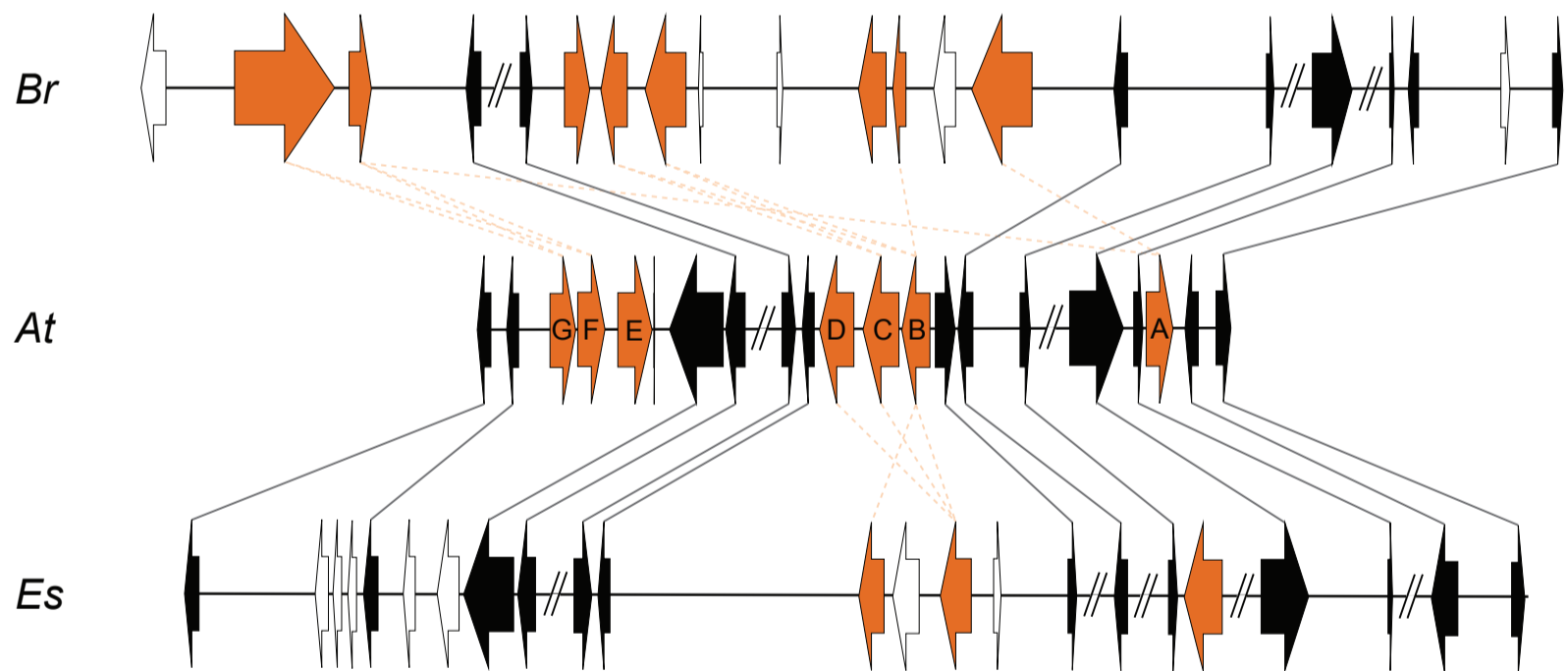

Supplement: Additional file 7: Figure S4. — Synteny in the RLM1 locus between five species. In (A) between A. lyrata (Al), A. thaliana (Col-0) (Al) and C. rubella (Cr) and (B) between B. rapa (Cr), A. thaliana (Col-0) and E. salsugineum (Es). The seven RLM1 genes; RLM1A (A, At1g64070), RLM1B, (B, At1g63880), RLM1C, (C, At1g63870), RLM1D, (D, At1g63860), RLM1E (E, At1g63750), RLM1F (F, At1g63740) and RLM1G (G, At1g63730) and the other TNL encoding genes in the four species are in orange (light orange if un-annotated). The non-TNL genes are depicted in black (synteny) or white (no synteny). Synteny between genes is depicted dotted lines showing similarity between two TNL proteins with an identity of 60 or higher. Reduction in bp length is depicted by the double forward slashes. [file 12870_2014_298_MOESM7_ESM.pdf]

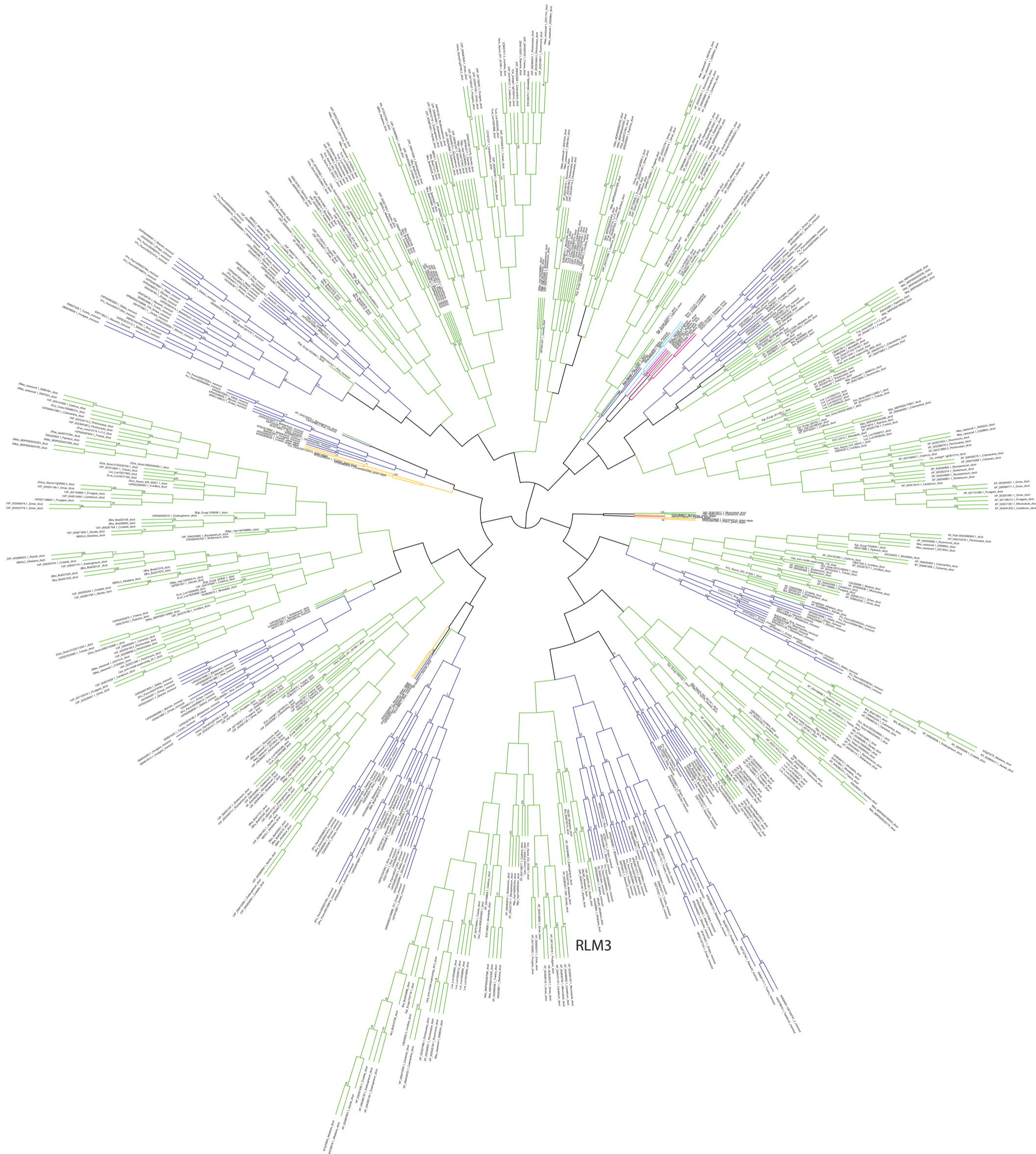

Supplement: Additional file 10: Figure S5. — Maximum likelihood analysis of the BRX domain. The GTR model was used and bootstrapping was with 1,000 replicates. Labeling is as follows: dicots (green), monocots (dark blue), green algae (orange), moss (pink) and lycophyta (light blue). The clades consisting of BRX domains of RLM3 is highlighted. [file 12870_2014_298_MOESM10_ESM.pdf]
