# Supplementary material for: Loss and retention of resistance genes in five species of the Brassicaceae family
Source: BMC Plant Biol. 2014 Nov 1;14:298. doi: 10.1186/s12870-014-0298-z (PMC4232680; doi:10.1186/s12870-014-0298-z)
Supplement: Additional file 5: Table S3. — Orthologous R genes between A. thaliana, A. lyrata, C. rubella, B. rapa and E. salsugineum. [file 12870_2014_298_MOESM5_ESM.docx]

**Table S3** Orthologous *R* genes between *A. thaliana* (At), *A. lyrata* (Al), *C. rubella* (Cr), *B. rapa* (Br) and Eutrema salsugineum (Es). The CNL genes are in green, TNL in dark blue and the TN genes in light blue. Conservation in the five species is in bold.

|  |  |  |  |  |  |  | Number of genes | | | | |
| --- | --- | --- | --- | --- | --- | --- | --- | --- | --- | --- | --- |
| **Clade** | **Structure** | **At** | **Al** | **Cr** | **Br** | **Es** | **At** | **Al** | **Cr** | **Br** | **Es** |
| 1 | CNL | At1g12210 | Al_CNL_31 |  |  |  | 1 | 1 | 0 | 0 | 0 |
| 2 | CNL | At1g50180 | Al_CNL_22 | Cr_CNL_33 |  |  | 1 | 1 | 1 | 0 | 0 |
| 3 | CNL | At3g14460 |  | Cr_CNL_35 |  |  | 1 | 0 | 1 | 0 | 0 |
| 4 | CNL | At3g46710  At3g46730  RPP13 |  |  | Br_CNL_39 |  | 3 | 0 | 0 | 1 | 0 |
| **5** | **CNL** | **ZAR1** | **Al_CNL_23** | **Cr_CNL_34** | **Br_CNL_6** | **Es_CNL_24** | **1** | **1** | **1** | **1** | **1** |
| 6 | CNL | At4g19050 | Al_CNL_38  Al_CNL_39 | Cr_TNL_33  Cr_CNL_41 |  | Es_CNL_28 | 1 | 2 | 2 | 0 | 1 |
| 7 | CNL | RPS2 | Al_CNL_11 |  | Br_CNL_22 | Es_CNL_19 | 1 | 1 | 0 | 1 | 1 |
| 8 | CNL | At4g27190 | Al_CNL_12 |  | Br_CNL_28 |  | 1 | 1 | 0 | 1 | 0 |
| 9 | CNL | At4g27220 |  |  | Br_CNL_30 |  | 1 | 0 | 0 | 1 | 0 |
| 10 | CNL |  |  | Cr_CNL_40 | Br_CNL_12 | Es_CNL_20 | 0 | 0 | 1 | 1 | 1 |
| 11 | CNL | ADR1-L1  ADR1-L2 | Al_CNL_28  Al_CNL_29 | Cr_CNL_37  Cr_CNL_38  Cr_CNL_39 | Br_CNL_41 |  | 2 | 2 | 3 | 1 | 0 |
| **12** | **CNL** | **At5g66900**  **At5g66910** | **Al_CNL_36** | **Cr_CNL_43** | **Br_CNL_8**  **Br_CNL_15**  **Br_CNL_42**  **Br_CNL_43** | **Es_CNL_25**  **Es_CNL_26** | **2** | **1** | **1** | **4** | **2** |
| 13 | CNL | At5g05400 | Al_CNL_10 |  |  |  | 1 | 1 | 0 | 0 | 0 |
| 14 | CNL | At5g47260 |  | Cr_CNL_11 |  |  | 1 | 0 | 1 | 0 | 0 |
| 15 | CNL |  |  |  | Br_CNL_26 | Es_CNL_3 | 0 | 0 | 0 | 1 | 1 |
|  |  |  |  |  |  | Total | 17 | 11 | 11 | 12 | 7 |
| 1 | TNL/TN |  | Al_TNL_89  Al_TNL_90 | Cr_TNL_32 | Br_TNL_19  Br_TNL_50  Br_TNL_108  Br_TN_7 | Es_TNL_43 | 0 | 2 | 1 | 4 | 1 |
| 2 | TNL |  | Al_TNL_88 |  |  | Es_TNL_42 | 0 | 1 | 0 | 0 | 1 |
| 3 | TNL/TN |  | Al_TN_6 | Cr_TNL_31 |  |  | 0 | 1 | 1 | 0 | 0 |
| 4 | TNL | At3g51560 | Al_TNL_85 |  |  |  | 1 | 1 | 0 | 0 | 0 |
| 5 | TNL | At4g12010 | Al_TNL_84 | Cr_TNL_29 | Br_TNL_23  Br_TNL_106 |  | 1 | 1 | 1 | 2 | 0 |
| **6** | **TNL** | **At4g19510** | **Al_TNL_83** | **Cr_TNL_28** | **Br_TNL_20** | **Es_TNL_40** | **1** | **1** | **1** | **1** | **1** |
| 7 | TNL |  | Al_TNL_77 | Cr_TNL_25 |  |  | 0 | 1 | 1 | 0 | 0 |
| 8 | TNL | At4g19530 |  | Cr_TNL_17 |  |  | 1 | 0 | 1 | 0 | 0 |
| **9** | **TNL** | **At5g45230** | **Al_TNL_80**  **Al_TNL_81**  **Al_TNL_82** | **Cr_TNL_26**  **Cr_TNL_27** | **Br_TNL_104** | **Es_TNL_38**  **Es_TNL_39** | **1** | **3** | **2** | **1** | **2** |
| 10 | TNL | **TTR1** | Al_TNL_79 |  |  |  | 1 | 1 | 0 | 0 | 0 |
| 11 | TNL | At4g36150 | Al_TNL_76 | Cr_TNL_21 | Br_TNL_8  Br_TNL_21 |  | 1 | 1 | 1 | 2 | 0 |
| 12 | TNL | At5g45200 | Al_TNL_75 |  |  | Es_TNL_37 | 1 | 1 | 0 | 0 | 1 |
| 13 | TNL |  | Al_TNL_74 | Cr_TNL_20 |  | Es_TNL_36 | 0 | 1 | 1 | 0 | 1 |
| 14 | TNL | **CSA1** | Al_TNL_73 |  |  | Es_TNL_34 | 1 | 1 | 0 | 0 | 1 |
| 15 | TNL | At3g51570 | Al_TNL_65 |  | Br_TNL_46 | Es_TNL_32 | 1 | 1 | 0 | 1 | 1 |
| 16 | TNL | At5g36930 | Al_TNL_58 |  |  |  | 1 | 1 | 0 | 0 | 0 |
| 17 | TNL | At1g27170 | Al_TNL_55  Al_TNL_56  Al_TNL_57 | Cr_TNL_16 | Br_TNL_13  Br_TNL_100  Br_TNL_101 | Es_TNL_30  Es_TNL_31 | 1 | 3 | 1 | 3 | 2 |
| **18** | **TNL** | **At5g17680** | **Al_TNL_54** | **Cr_TNL_13** | **Br_TNL_96**  **Br_TNL_97** | **Es_TNL_28** | **1** | **1** | **1** | **2** | **1** |
| 19 | TNL |  | Al_TNL_53 |  | Br_TNL_93 |  | 0 | 1 | 0 | 1 | 0 |
| 20 | TNL | At1g17600  At5g40100 | Al_TNL_50  Al_TNL_51 | Cr_TNL_10  Cr_TNL_11 | Br_TNL_33  Br_TNL_52 |  | 2 | 2 | 2 | 2 | 0 |
| 21 | TNL | At1g63740  At1g63730  At5g58120 | Al_TNL_33  Al_TNL_35 |  | Br_TNL_14  Br_TNL_26  Br_TNL_80  Br_TNL_81  Br_TNL_82 |  | 3 | 2 | 0 | 5 | 0 |
| 22 | TNL |  | Al_TNL_31 |  |  | Es_TNL_20 | 0 | 1 | 0 | 0 | 1 |
| 23 | TNL | **ADR2**  At1g56520  At1g56540  At1g66090 | Al_TNL_30 |  |  | Es_TNL_16  Es_TNL_17 | 4 | 1 | 0 | 0 | 2 |
| **Clade** | **Structure** | **At** | **Al** | **Cr** | **Br** | **Es** | **At** | **Al** | **Cr** | **Br** | **Es** |
| 24 | TNL |  | Al_TNL_40 |  |  | Es_TNL_23 | 0 | 1 | 0 | 0 | 1 |
| 25 | TNL | At2g16870 | Al_TNL_37 |  |  |  | 1 | 1 | 0 | 0 | 0 |
| 26 |  | At5g40910  At5g41540  At5g41550  At5g41740  At5g41750 | Al_TNL_41  Al_TNL_42  Al_TNL_43  Al_TNL_44  Al_TNL_45  Al_TNL_46  Al_TNL_47 |  | Br_TNL_89  Br_TNL_90  Br_TNL_91  Br_TNL_92 | Es_TNL_24  Es_TNL_25  Es_TNL_26 | 5 | 7 | 0 | 4 | 3 |
| 27 | TNL |  | Al_TNL_23 |  | Br_TNL_73 |  | 0 | 1 | 0 | 1 | 0 |
| 28 | TNL/TN |  |  |  | Br_TNL_71  Br_TN_2 | Es_TNL_12 | 0 | 0 | 0 | 2 | 1 |
| 29 | TNL | **RPS6** | Al_TNL_20 |  |  |  | 1 | 1 | 0 | 0 | 0 |
| 30 | TNL | **TAO** | Al_TNL_17 |  |  | Es_TNL_9 | 1 | 1 | 0 | 0 | 1 |
| 31 | TNL | At5g18350  At5g18370 |  |  | Br_TNL_6  Br_TNL_17  Br_TNL_18  Br_TNL_44  Br_TNL_66  Br_TNL_67  Br_TNL_68  Br_TNL_69  Br_TNL_70 | Es_TNL_11 | 2 | 0 | 0 | 9 | 1 |
| 32 | TNL | At5g11250 | Al_TNL_1 |  | Br_TNL_16  Br_TNL_42  Br_TNL_45  Br_TNL_58  Br_TNL_59 | Es_TNL_4 | 1 | 1 | 0 | 5 | 1 |
| 33 | TNL | At4g16900  At4g16920  At4g16940  **RPP4**  **RPP5**  **SNC1** | Al_TNL_29 | Cr_TNL_7 |  |  | 6 | 1 | 1 | 0 | 0 |
| 34 | TNL | At4g11170 |  |  |  | Es_TNL_27 | 1 | 0 | 0 | 0 | 1 |
| 35 | TNL | At4g08450 | Al_TNL_25 |  |  |  | 1 | 1 | 0 | 0 | 0 |
| 36 | TNL | At3g44400  At3g44630  At3g44670  **RPP1** | Al_TNL_12  Al_TNL_13 |  |  |  | 4 | 2 | 0 | 0 | 0 |
| 37 | TNL | At1g69550 |  | Cr_TNL_1  Cr_TNL_2  Cr_TNL_3 |  |  | 1 | 0 | 3 | 0 | 0 |
| 38 | TNL | At1g31540  At5g46260  At5g46270 | Al_TNL_26  Al_TNL_27 | Cr_TNL_5 |  |  | 3 | 2 | 1 | 0 | 0 |
| 39 | TNL | At1g65850  At5g38340 | Al_TNL_6  Al_TNL_7  Al_TNL_8  Al_TNL_9  Al_TNL_10 |  |  |  | 2 | 5 | 0 | 0 | 0 |
| 40 | TNL | At5g18360 | Al_TNL_49 |  | Br_TNL_2 |  | 1 | 1 | 0 | 1 | 0 |
| 41 | TNL | At5g17970 | Al_TNL_48 |  | Br_TNL_12  Br_TNL_27 |  | 1 | 1 | 0 | 2 | 0 |
| 42 | TNL |  |  | Cr_TNL_14  Cr_TNL_15 | Br_TNL_1  Br_TNL_98  Br_TNL_99 | Es_TNL_29 | 0 | 0 | 2 | 3 | 1 |
|  |  |  |  |  |  | Total | 53 | 55 | 21 | 51 | 25 |
| 1 | TN | At1g72850  At4g09420 |  |  | Br_TN_15 |  | 2 | 0 | 0 | 1 | 0 |
| 2 | TN/TNL | At1g72890  At1g72900  At1g72910  At1g72940  At1g72950 |  | Cr_TN_1  Cr_TNL_12 | Br_TN_3  Br_TN_8  Br_TN_14 |  | 5 | 0 | 2 | 3 | 0 |
| 3 | TN | **RLM3** | Al_TN_4 |  |  |  | 1 | 1 | 0 | 0 | 0 |
| 4 | TN |  | Al_TN_5 |  | Br_TN_1  Br_TN_4  Br_TN_5 |  | 0 | 1 | 0 | 3 | 0 |
| 5 | TN |  | Al_TN_7 |  | Br_TN_11 |  | 0 | 1 | 0 | 1 | 0 |
|  |  |  |  |  |  | Total | 8 | 3 | 2 | 8 | 0 |
