# Supplementary material for: Loss and retention of resistance genes in five species of the Brassicaceae family
Source: BMC Plant Biol. 2014 Nov 1;14:298. doi: 10.1186/s12870-014-0298-z (PMC4232680; doi:10.1186/s12870-014-0298-z)
Supplement: Additional file 8: Table S4. — Distribution of presence and absence of gene members in the RLM1 locus in 19 A. thaliana accessions. [file 12870_2014_298_MOESM8_ESM.docx]

**Table S4** Distribution of presence and absence of gene members in the *RLM1* locus in 19 *A. thaliana* accessions.

|  | AT1G63730 | AT1G63740 | AT1G63750 | AT1G63860 | AT1G63870 | AT1G63880 | AT1G64070 |
| --- | --- | --- | --- | --- | --- | --- | --- |
|  | *RLM1G* | *RLM1F* | *RLM1E* | *RLM1D* | *RLM1C* | *RLM1B* | *RLM1A* |
| Col-0 | TNL | TNL | TNL | T(N)L | TNL | TNL | TNL |
| Bur-0 | TNL | TNL | TNL | L | - | - | TN |
| Can-0 | TNL | TNL | TNL | T(N) | (N)L | TNL | TNL |
| Ct-1 | TNL | TNL | TNL | L | L | - | TNL |
| Edi-0 | TNL | TNL | TNL | T(N) | (N)L | TNL | TNL |
| Hi-0 | TNL | NL | TNL | L | TNL | L | TNL |
| Kn-0 | TNL | TNL | TNL | T(N) | L | TNL | L |
| Ler-0 | TNL | TNL | TNL | L | TN | L | TNL |
| Mt-0 | TNL | TNL | TNL | TN | - | TNL | TNL |
| No-0 | TNL | TNL | TNL | L | TNL | - | TNL |
| Oy-0 | TNL | TNL | TNL | T(N) | L | TN | T |
| Po-0 | TNL | TNL | TNL | L | TNL | (N) | TNL |
| Rsch-4 | TNL | TNL | TNL | L | TN | TNL | TN |
| Sf-2 | TNL | L | (T)NL | T(N) | TNL | TN | TNL |
| Tsu-0 | TNL | TNL | TNL | L | (T)NL | (N)L | TNL |
| Wil-2 | TNL | TNL | TNL | L | TN | TNL | TNL |
| Ws-0 | TNL | TNL | TNL | - | TNL | TNL | TNL |
| Wu-0 | TNL | TNL | TNL | T(N)L | TNL | TNL | TNL |
| Zu-0 | TNL | TNL | TNL | L | - | TN | TNL |
| Sum | 19 | 17 | 18 | 2 | 7 | 9 | 15 |
