# Supplementary material for: Loss and retention of resistance genes in five species of the Brassicaceae family
Source: BMC Plant Biol. 2014 Nov 1;14:298. doi: 10.1186/s12870-014-0298-z (PMC4232680; doi:10.1186/s12870-014-0298-z)
Supplement: Additional file 9: Table S5. — p-distance of the different TNL encoding genes in the RLM1 locus in the 19 A. thaliana accessions. [file 12870_2014_298_MOESM9_ESM.docx]

**Table S5** *p-*distance of the different *TNL* encoding proteins in the *RLM1* locus in the 19 *A. thaliana* accessions.

|  | AT1G63730 | AT1G63740 | AT1G63750 | AT1G63860 | AT1G63870 | AT1G63880 | AT1G64070 | Average |  |
| --- | --- | --- | --- | --- | --- | --- | --- | --- | --- |
|  | *RLM1G* | *RLM1F* | *RLM1E* | *RLM1D* | *RLM1C* | *RLM1B* | *RLM1A* |  |  |
| Bur-0 | 0.6 | 1.0 | 0.7 | 0.6 | - | - | 0.6 | 0.7 | absent |
| Can-0 | 0.4 | 0.6 | 0.6 | 11.3 | 4.3 | 6.2 | 0.5 | 3.4 | 0-1% |
| Ct-1 | 0.6 | 0.9 | 0.8 | 0.6 | 5.9 | - | 0.6 | 1.6 | >1-5% |
| Edi-0 | 0.4 | 0.1 | 0.1 | 10.3 | 4.9 | 6.4 | 1.1 | 3.3 | >5-10% |
| Hi-0 | 0.7 | 0.6 | 0.9 | 0.4 | 0.4 | 2.4 | 0.7 | 0.9 | >10% |
| Kn-0 | 0.7 | 1.1 | 0.7 | 11.6 | 2.6 | 5.9 | 3.8 | 3.8 |  |
| Ler-0 | 0.3 | 0.2 | 0.6 | 0.4 | 5.6 | 9.3 | 0.5 | 2.4 |  |
| Mt-0 | 0.3 | 0.6 | 1.2 | 0.4 | - | 4.2 | 0.2 | 1.2 |  |
| No-0 | 1.4 | 0.9 | 0.7 | 0.6 | 5.0 | - | 0.3 | 1.5 |  |
| Oy-0 | 0.1 | 0.2 | 1.0 | 12.2 | 5.7 | 3.3 | 9.0 | 4.5 |  |
| Po-0 | 0.1 | 0.2 | 1.4 | 0.4 | 0.4 | 1.8 | 0.6 | 0.7 |  |
| Rsch-4 | 0.2 | 0.8 | 1.2 | 0.3 | 0.4 | 4.8 | 0.6 | 1.2 |  |
| Sf-2 | 0.6 | 0.5 | 0.8 | 10.1 | 2.5 | 2.5 | 0.5 | 2.5 |  |
| Tsu-0 | 0.3 | 0.4 | 0.7 | 0.4 | 4.5 | 7.3 | 0.9 | 2.1 |  |
| Wil-2 | 1.2 | 1.1 | 1.0 | 3.8 | 0.2 | 2.3 | 0.4 | 1.4 |  |
| Ws-0 | 0.2 | 0.3 | 0.7 | - | 4.4 | 4.6 | 13.8 | 4.0 |  |
| Wu-0 | 0.0 | 0.1 | 0.1 | 0.0 | 0.0 | 0.0 | 0.9 | 0.2 |  |
| Zu-0 | 1.3 | 0.8 | 0.8 | 0.6 | - | 2.9 | 0.9 | 1.2 |  |
| Average | 0.5 | 0.6 | 0.8 | 3.8 | 3.1 | 4.3 | 2.0 | 2.0 |  |
|  |  |  |  |  |  |  |  |  |  |
| Ka/Ks ratio | 0.0-3.0 | 0.0-3.0 | 0.9-3.0 | 0.0-4.7 | 0.0-3.0 | 0-4.2 | 0.0-7.0 |  |  |
| Average | 0.8 | 1.1 | 1.6 | 3.3 | 1.8 | 2.2 | 3.1 |  |  |
